# Enhancing Early Detection of Oral Squamous Cell Carcinoma: A Deep Learning Approach with LRT-Enhanced EfficientNet-B3 for Accurate and Efficient Histopathological Diagnosis

**DOI:** 10.3390/diagnostics15131678

**Published:** 2025-07-01

**Authors:** A. A. Abd El-Aziz, Mahmood A. Mahmood, Sameh Abd El-Ghany

**Affiliations:** Department of Information Systems, College of Computer and Information Sciences, Jouf University, Sakaka 72388, Saudi Arabia; mamahmood@ju.edu.sa (M.A.M.); saabdelwahab@ju.edu.sa (S.A.E.-G.)

**Keywords:** oral tumors, squamous cell carcinoma, deep learning, histopathological images, EfficientNet-B3, learning rate tuning, oral tumor dataset, multi-cancer dataset

## Abstract

**Background/Objectives:** Oral cancer, particularly oral squamous cell carcinoma (OSCC), ranks as the sixth most prevalent cancer globally, with rates of occurrence on the rise. The diagnosis of OSCC primarily depends on histopathological images (HIs), but this method can be time-intensive, expensive, and reliant on specialized expertise. Manual diagnosis often leads to inaccuracies and inconsistencies, highlighting the urgent need for automated and dependable diagnostic solutions to enhance early detection and treatment success. **Methods:** This research introduces a deep learning (DL) approach utilizing EfficientNet-B3, complemented by learning rate tuning (LRT), to identify OSCC from histopathological images. The model is designed to automatically modify the learning rate based on the accuracy and loss during training, which improves its overall performance. **Results:** When evaluated using the oral tumor dataset from the multi-cancer dataset, the model demonstrated impressive results, achieving an accuracy of 99.84% and a specificity of 99.92%, along with other strong performance metrics. **Conclusions:** These findings indicate its potential to simplify the diagnostic process, lower costs, and enhance patient outcomes in clinical settings.

## 1. Introduction

Cancer, characterized by uncontrolled cell growth damaging tissues, is increasing in prevalence across all demographics. This rising trend underscores the critical need for effective screening, early detection, and timely treatment. Consequently, awareness programs and clinical protocols have been developed to help reduce the disease’s impact [1]. According to the 2020 report by the World Health Organization (WHO), cancer is responsible for approximately 10 million deaths worldwide each year. The report indicated that low- and lower-middle-income countries account for around 30% of cancer cases linked to infections such as human papillomavirus (HPV) and hepatitis [2].

**Figure 1:**
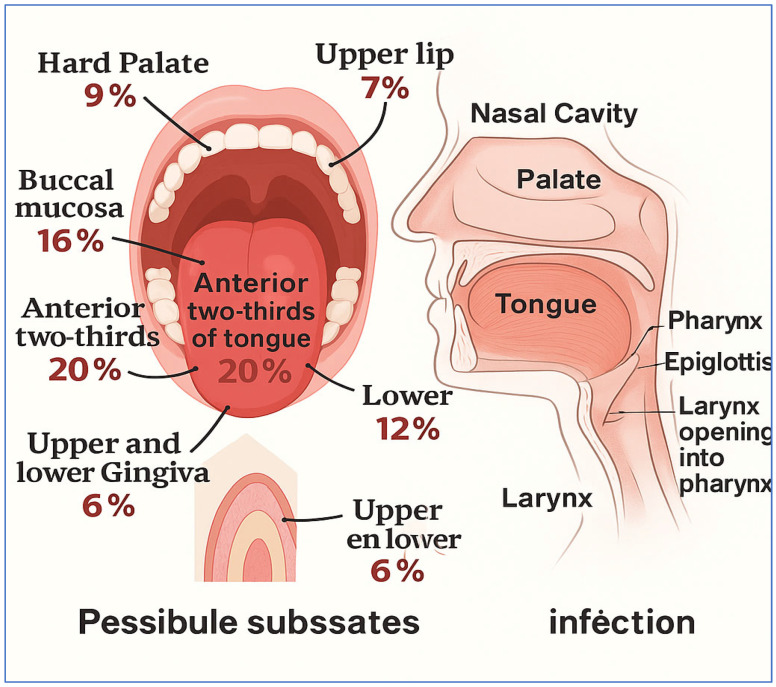
A summary of the head and neck regions, along with possible subsites that may be affected by oral cancer infections [8].

Oral cancer is identified as the sixth most common type of cancer globally and falls under the broader category of head and neck cancers. Each year, it is estimated that there will be about 475,000 new cases of oral cancer diagnosed, which is a specific subtype of head and neck cancer [3]. Statistics indicate that approximately 80% of patients will experience the early stages of the disease, while only about 20% will survive the more advanced stages [4]. Oral cancer leads to the formation of cancerous cells in the lips and various regions of the oral cavity. In 2020, there were 377,713 new cases of lip and oral cavity cancer, leading to 177,757 deaths [5]. It is recognized as the fifteenth leading cause of death among different cancer types worldwide, with at least four individuals affected for everyone hundred thousand people globally [6,7]. Figure 1 shows a summary of the head and neck regions, along with possible subsites that may be affected by oral cancer infections:

The most common types of oral cancer include OSCC, verrucous carcinoma, carcinomas of minor salivary glands, lymphoma, and mucosal melanoma. Among these, OSCC is the most significant, representing approximately 84–97% of all oral cancer cases [9]. OSCC arises from epithelial dysplasia that develops from precursor lesions known as potentially malignant oral disorders (PMODs). Conditions, such as leukoplakia, oral lichen planus, and erythroplakia, can progress to malignant tumors if they remain undiagnosed and untreated [10]. Research shows that PMODs can present both homogeneous and heterogeneous symptoms. If heterogeneous symptoms are not identified quickly, they are more likely to evolve into malignant lesions [11]. An international research agency has forecast an increase in cancer patients, from 1 million in 2012 to over 1.7 million by 2035. This trend indicates that the death rate may also rise, increasing from 680,000 to between 1 million and 2 million by 2035 [12]. OSCC can affect individuals of various ages, but it is more commonly observed in older adults and those with specific risk factors, such as [13,14] **tobacco use** (smoking cigarettes, cigars, pipes, or using tobacco to chew significantly increases the risk), **heavy alcohol consumption** (drinking alcohol excessively, particularly when combined with tobacco use, is a major risk factor), **HPV** (infection with certain types of HPV, particularly HPV-16, is associated with a higher risk of oropharyngeal cancers), **sun exposure** (prolonged exposure to the sun can lead to cancer on the lips, especially the lower lip), and **poor oral hygiene and diet** (a diet lacking fruits and vegetables and poor oral care may increase the risk).

Due to increasing consumption of tobacco and alcohol, the rates of OSCC have significantly escalated in recent years in certain areas, like Melanesia, South-Central Asia, and Central and Eastern Europe. This trend has also extended to such countries as Australia, New Zealand, and North America, though the impact is comparatively smaller [15].

HIs of OSCC exhibit specific characteristics. Consequently, specialized processing techniques are necessary for the accurate diagnosis and prediction of cancer type based on these OSCC images. There are three types of OSCC based on histopathology [16]. **Well-differentiated OSCC** demonstrates structured growth patterns, distinct keratin pearls, and a relatively low level of pleomorphism. The tumor cells closely resemble normal squamous cells. **Moderately differentiated OSCC** shows some level of organization, with decreased keratin production and a moderate presence of cellular atypia. Keratin pearls are less common in this differentiation. **Poorly differentiated OSCC** is characterized by a chaotic growth pattern, minimal to no keratinization, a high degree of pleomorphism, and numerous atypical mitotic figures. The cells exhibit significant differences from normal squamous cells.

The symptoms associated with OSCC can differ based on the cancer’s location and progression but commonly include the following [17]: **chronic mouth ulcer** (an ulcer in the mouth or on the lips that persists for more than a few weeks. This is frequently one of the initial indicators), **pain or discomfort** (ongoing pain or discomfort within the mouth that may intensify during chewing, swallowing, or speaking), **lumps or thickening** (a lump, thickened area, or rough texture in the cheeks, gums, or other parts of the mouth), **red or white spot** (red or white spots on the gums, tongue, tonsils, or the inner lining of the mouth. These spots, referred to as erythroplakia and leukoplakia, respectively, can occasionally develop into cancer), and **numbness or discomfort** (numbness or unusual pain in any region of the face, mouth, or neck, including the tongue and jaw).

Early detection of OSCC is critical due to its high mortality rate. While OSCC lacks distinct early clinical signs, predictive indicators include lesion location, color, size, appearance, and tobacco/alcohol use [18]. Survival rates starkly illustrate the importance of early diagnosis: the 5-year survival rate is ~85% for early-stage OSCC but drops to ~40% for advanced stages [19,20]. This significant survival difference underscores the urgent need for diagnostic tools capable of identifying OSCC in its initial malignant stages.

Key diagnostic methods for OSCC include various techniques beyond physical exams, such as endoscopy, liquid biopsies, imaging (ultrasound, MRI, CT), spectroscopy, and biomarker detection [9]. However, histopathological analysis remains the gold standard. While it can be invasive and time-consuming, histopathology differentiates malignant tumors from benign tumors by examining tissue structure and cellular changes microscopically. It tracks cancer progression from dysplasia to invasion, assessing cell proliferation, abnormal growth, cytoplasmic/cellular atypia, epithelial alterations, and deep tissue cytoarchitecture [8,21].

Visual inspection for potentially malignant oral disorders (HIs) is subjective and prone to errors, showing lower sensitivity and specificity than computerized tools [17]. Human inspection biases can cause false positives (reducing specificity) or false negatives (reducing sensitivity). Therefore, computer-aided diagnosis (CAD) systems are essential for automatically differentiating benign from malignant OSCC cells. By utilizing tumor characteristics, CAD enhances classification accuracy, aiding clinicians in decision-making and minimizing errors.

Artificial intelligence (AI), particularly machine learning (ML) and DL, shows significant promise for improving diagnostic processes, especially in oral cancer, by offering innovative solutions to disease identification and prognosis prediction [22]. DL, a subset of ML using multi-layered artificial neural networks (ANNs), effectively diagnoses biomedical images by learning from large labeled datasets, eliminating the need for manual feature engineering required by traditional methods [23,24]. Convolutional neural networks (CNNs), a prominent DL technique, demonstrate high efficiency in image classification tasks [25,26]. A key advantage of CNNs is their effectiveness even when pre-trained on different tasks (transfer learning) [27,28].

DL models are vital for medical image analysis, performing comparably to or better than human specialists in identifying features for automated cancer lesion detection and classification [29]. To optimize artificial neural networks (ANNs), especially convolutional neural networks (CNNs), for specific applications, like cancer detection, careful adjustment of hyperparameters—particularly the learning rate (LR)—is crucial for improving accuracy. The optimal LR is often found through trial and error, commonly using a constant learning rate approach [30].

Optimal LR selection is crucial for efficient model training. A too-low LR slows convergence, requiring more iterations, while a too-high LR causes instability and poor results. An optimal LR speeds convergence and critically influences the backpropagated loss gradient’s magnitude, affecting the model’s ability to find global minima (only computational effort beyond a local minimum is wasteful).

**Figure 2:**
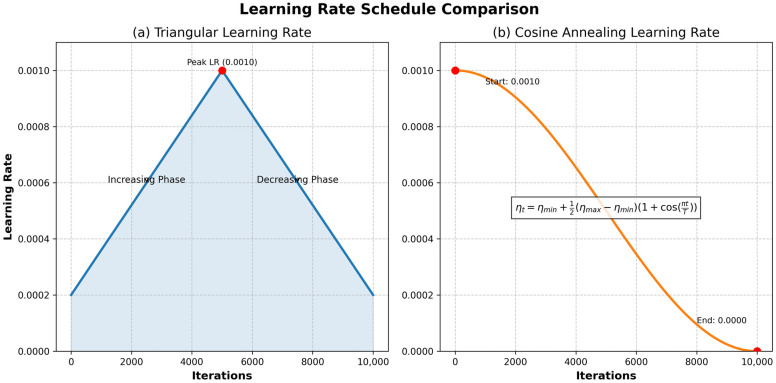
The LR varies between the minimum and maximum thresholds shown in figures (**a**,**b**). It continues this cycle until it finishes the final epoch [33].

LRT adapts the LR (e.g., reducing it after accuracy plateaus or when stuck in local minima), unlike non-adaptive schedules that remain constant or decay rigidly. Common decay methods include inverse square root, exponential, linear, and cosine decay, often requiring trial and error. LRT is integrated with gradient descent algorithms (GDA). Effective LRT algorithms include cyclical learning rate (CLR) [30], stochastic gradient descent with warm restarts (SGDWR) [31], and stochastic weight averaging (SWA) [32]. LR variations are illustrated in Figure 2.

This research proposes a robust fine-tuned model based on EfficientNet-B3 that focuses on the classification of HIs into two distinct categories, namely normal and OSCC. Features from the HIs are obtained by utilizing pre-trained weights from the EfficientNet-B1, EfficientNet-B2, EfficientNet-B3, DenseNet169, and InceptionV3 models. The model uses an innovative LR adjustment technique known as the LRT. This method automatically adjusts the LR at the start of each epoch by analyzing the training accuracy and loss metrics from the previous epoch. Hence, the proposed model will make it easier for clinicians to catch OSCC tumors early and will reduce the time and money it takes to diagnose them. The proposed model was evaluated on the oral tumor dataset from the multi-cancer dataset containing 1224 oral HIs of normal and OSCC cells, captured at magnifications of 100× and 400×. We preprocessed the HIs by augmentation, resizing, and normalization techniques. Moreover, we split the oral tumor dataset into three parts, with 70% for a training set (6998 HIs), 15% for a test set (1501 HIs), and 15% for a validation set (1501 HIs). The proposed fine-tuned classifier was evaluated and compared with traditional classifiers. A summary of our research’s contributions is outlined below:

- We developed a refined model utilizing the EfficientNet-B3 approach for the detection of OSCC tumors by analyzing the HI oral cancer data from the oral tumor dataset.
- The LRT algorithm was employed to automatically make adjustments at the beginning of each epoch by evaluating the training accuracy and loss metrics from the preceding epoch.
- An ablation study was performed to determine the best hyperparameters. The refined model that was created assists healthcare providers in comprehending the essential elements that lead to oral tumors.
- Our enhanced technique results in improved outcomes by effectively recognizing patient conditions, thereby streamlining the diagnostic process. This innovation provides radiologists with an automated tool for assessing OSCC detection, which minimizes the risk of misdiagnosis.
- The newly designed CNN model was meticulously created to strike a suitable balance and mitigate issues related to overfitting.
- An extensive statistical evaluation was conducted using all available methodologies.
- When compared to four DL models (EfficientNet-B1, EfficientNet-B2, DenseNet169, and InceptionV3), The EfficientNet-B3 model exhibited exceptional performance in managing binary classification tasks.
- The proposed model reached an accuracy of 99.84%, specificity of 99.92%, precision of 97.94%, recall of 97.93%, and an F1-score of 97.93%.

The subsequent sections of this paper are structured as follows: Section 2 provides a review of the current literature regarding diagnostic systems for OSCC. Section 3 details the preprocessing techniques utilized on the HIs of oral cancer drawn from the oral tumor dataset, along with the methodology of our proposed model. Section 4 presents the experimental results obtained from implementing the proposed framework. Section 5 concludes with a discussion of our proposed model.

## 2. Literature Review

The diagnosis of OSCC tumors is a significant area of research within medical image analysis. Numerous studies approach this issue from various angles, contributing to a diverse array of perspectives on the topic. For example, Shetty, S.K. et al. [3] introduced an innovative model for classification, known as the Duck Pack Optimization with Deep Transfer Learning (DPODTL-OSC3). This model utilized HIs to improve classification accuracy and effectively differentiate between normal and cancerous tissues. To accomplish this, a variational autoencoder (VAE) was employed for the detection and classification of OSCC. The effectiveness of the DPODTL-OSC3 model was assessed through performance validation and comparative analysis using a database of HIs. The DPODTL-OSC3 model achieved an impressive accuracy score of 97.28%.

Nagarajan, B. et al. [17] proposed a DL framework including an intermediary layer positioned between the feature extraction layers and the classification layers. This framework aimed to classify HIs into two categories, namely normal and OSCC. The intermediary layer employed a newly introduced swarm intelligence technique called the modified gorilla troops optimizer. Three established CNN architectures—InceptionV2, MobileNetV3, and EfficientNetB3—were examined for feature extraction. The classification layers consist of two fully connected neural network layers, which incorporate batch normalization and dropout methods. Among the evaluated feature extraction models, MobileNetV3 achieved the highest accuracy of 0.89, showcasing robust performance. When the modified gorilla troops optimizer was integrated as an intermediary layer, the accuracy improved to 0.95.

Ahmed, I.A. et al. [34] developed several hybrid models that utilized fused CNN features to diagnose OSCC cancer using the OSCC-100× and OSCC-400× datasets. The first approach relied on pre-trained models, such as GoogLeNet, ResNet101, and VGG16, but the results were not satisfactory. The second approach also employed GoogLeNet, ResNet101, and VGG16, this time integrating the adaptive region growing (ARG) segmentation algorithm. The third approach combined GoogLeNet, ResNet101, and VGG16 with ANN and XGBoost networks, leveraging the ARG hashing algorithm. The fourth strategy for diagnosing oral cancer used ANN and XGBoost, concentrating on features fused from CNN models. The ANN that incorporated features from GoogLeNet, ResNet101, and VGG16 achieved an area under the curve (AUC) of 98.85%, an accuracy of 99.3%, a sensitivity of 98.2%, a precision of 99.5%, and a specificity of 98.35%.

Das, M. et al. [14] introduced a CNN model for the early and automatic detection of OSCC. For experimental validation, HIs of oral cancer were utilized. The proposed model was compared and evaluated against advanced DL architectures, including VGG16, VGG19, AlexNet, ResNet50, ResNet101, MobileNet, and InceptionNet. The model achieved a cross-validation accuracy of 97.82%.

Panigrahi, S.et al. [35] developed an effective CAD system developed based on DL for classifying OSCC HIs using two proposed methods. The first method involved employing transfer learning (TL) with deep convolutional neural networks (DCNNs) to identify the most suitable model for differentiating between benign and malignant tumors. To tackle the challenge of a limited dataset and improve the training efficiency of the proposed model, several pre-trained models—VGG16, VGG19, ResNet50, InceptionV3, and MobileNet—were fine-tuned by training half of their layers while keeping the others fixed. The second method introduced a baseline DCNN architecture that was trained from scratch, consisting of 10 convolutional layers. Additionally, a comparative analysis of these models was carried out, concentrating on classification accuracy and various performance metrics. The experimental results indicated that ResNet50 significantly surpassed the selected fine-tuned DCNN models and the baseline model, achieving an accuracy of 96.6%, along with precision and recall values of 97% and 96%, respectively.

Fati, S.M. et al. [36] focused on achieving effective early diagnosis of OSCC through the implementation of hybrid techniques that utilize fused features. The first proposed method combined CNN models, specifically AlexNet and ResNet-18, with the support vector machine (SVM) algorithm. This hybrid approach had shown exceptional performance in diagnosing the OSCC dataset. The second method involved extracting hybrid features using the same CNN models (AlexNet and ResNet-18), integrated with color, texture, and shape features. These additional features were derived from several algorithms, including the fuzzy color histogram (FCH), discrete wavelet transform (DWT), local binary pattern (LBP), and gray-level co-occurrence matrix (GLCM). To manage the high dimensionality of the dataset features, principal component analysis (PCA) was used to reduce dimensions before inputting the data into an ANN for diagnosis, which resulted in promising accuracy. All proposed systems produced outstanding results in the histological diagnosis of OSCC. Notably, the ANN model that utilized hybrid features from AlexNet, along with DWT, LBP, FCH, and GLCM, achieved an accuracy rate of 99.1%, a specificity of 99.61%, a sensitivity of 99.5%, a precision of 99.71%, and an area under the curve (AUC) of 99.52%.

Afify, H.M. et al. [37] introduced an innovative model that utilized deep transfer learning (TL) to predict OSCC from HIs. The model incorporated gradient-weighted class activation mapping (Grad-CAM) techniques to identify and highlight the lesion areas within the images. Using a modern public dataset, the model analyzed 1224 oral HIs featuring both normal and OSCC cells at magnifications of 100× and 400×. The primary objective was to implement ten different algorithms using CNN to develop a predictive model for HIs of OSCC, enhancing the accuracy of classifying the images as either malignant or normal. After evaluating the performance of these models, the results were compared to determining which model performed the best. The secondary focus was on the Grad-CAM validation method, which was used to pinpoint the lesion area in an OSCC image based on the model with the highest performance. This validation step was essential, as it significantly affected the overall robustness of the prediction model for OSCC HIs. The experimental results showed that ResNet-101 achieved an impressive accuracy of 100% at a magnification of 100×, while EfficientNet-B0 recorded a high accuracy of 95.65% at 400× magnification.

Albalawi, E. et al. [38] utilized a database for analyzing oral cancer, containing 1224 images from 230 patients. These images were taken at various magnifications and were used to create a specialized DL model based on the EfficientNet-B3 architecture. The model was trained to distinguish between normal epithelium and OSCC tissues. It employed advanced techniques, such as data augmentation, regularization methods, and optimization strategies. The customized DL model achieved remarkable success, reaching an accuracy of 99% during testing with the dataset. This outstanding accuracy underscored the model’s effectiveness in accurately differentiating between healthy epithelium and OSCC tissues.

Badawy, M. et al. [8] introduced an innovative empirical framework to enhance the precise and automatic classification of oral cancer by analyzing microscopic histopathology slide images. The system utilized CNNs and improved performance through TL. Furthermore, it incorporated advanced optimization algorithms, specifically the aquila optimizer (AO) and gorilla troops optimizer (GTO), both of which were cutting-edge metaheuristic techniques. This unique approach effectively addressed the biases and uncertainties commonly encountered during the preprocessing and optimization phases. In the experiments conducted, the effectiveness of several prominent pre-trained TL models was assessed, including VGG19, VGG16, MobileNet, MobileNetV3Small, MobileNetV2, MobileNetV3Large, NASNetMobile, and DenseNet201. These models were initialized using weights derived from the ImageNet dataset. The experiments utilized the Histopathologic Oral Cancer Detection dataset, which features a normal category with 2494 images and an OSCC category containing 2698 images. The results revealed a performance difference between the AO and GTO, with the AO consistently delivering superior outcomes across all models, except for the Xception model. Remarkably, the DenseNet201 model stood out as the most accurate, achieving an impressive average accuracy of 99.25% with the AO and 97.27% with the GTO.

Ananthakrishnan, B. et al. [39] focused on differentiating between normal and cancerous cells in the oral cavity using two distinct methods to achieve high accuracy. The first method involved extracting local binary patterns and metrics from a histogram derived from the dataset. These features were then fed into various ML models. The second approach was hybrid in nature, combining neural networks as the primary feature extractor with a random forest (RF) classifier. It involved extracting image features through pre-trained CNNs and training a classification model using the resulting feature vectors. This strategy allowed the research to bypass the extensive datasets typically needed for training DL models by leveraging features from a pre-trained CNN model to train the random forest classifier. The research utilized a dataset of 1224 images, which were divided into two groups with different resolutions. The model’s performance was evaluated based on accuracy, specificity, sensitivity, and the AUC. The findings revealed that the proposed methodology achieved a maximum test accuracy of 96.94% and an AUC of 0.976 using 696 images at 400× magnification. Additionally, it reached a peak test accuracy of 99.65% and an AUC of 0.9983 with only 528 images at 100× magnification.

The limitations of the previous studies are as follows:

1. The authors of the previously mentioned research used a fixed LR, while our proposed model implemented the LRT to overcome the limitations of a fixed LR. The LRT dynamically adjusts the LR during training based on various factors, such as gradient magnitudes, loss convergence, and training progress. This flexibility allows the model to navigate complex optimization landscapes more efficiently. By adapting the LR in real time, the LRT facilitates faster convergence, accelerating progress during the initial training phases and reducing oscillations or overshooting as optimization approaches convergence. Consequently, our proposed model achieved higher accuracy compared to the current state-of-the-art approaches in this field.
2. The authors of the previously mentioned research did not conduct an ablation study. In contrast, we performed this study to understand the effects of individual components or features within our proposed model by systematically removing or modifying them and observing the impact on performance.
3. The authors of the previously mentioned research did not use statistical evaluation, while we implemented an extensive statistical evaluation.

## 3. Materials and Methods

### 3.1. Multi-Cancer and Oral Tumor Datasets

The multi-cancer dataset is a comprehensive collection of various types of cancer intended for research and analysis. It includes 8 main cancer categories and 26 subcategories, making it a significant resource for medical image classification and DL projects. This dataset is sourced from eight different cancer datasets, including those pertaining to oral cancer [40,41].

**Figure 3:**
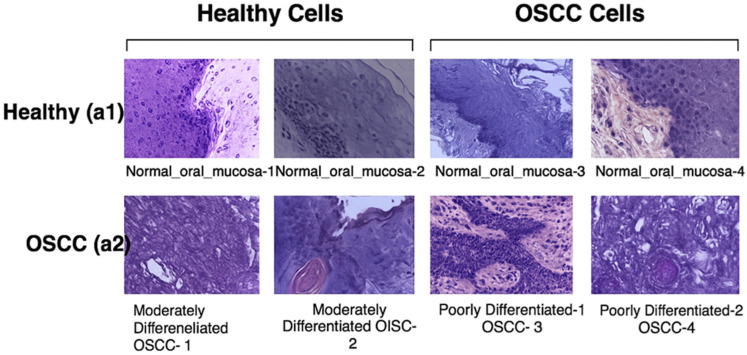
Images show healthy cells and OSCC cells [43].

The oral tumor dataset contains a total of 1224 images organized into two sets with varying resolutions. The first set has 89 HIs showing the normal epithelium of the oral cavity and 439 images of OSCC captured at 100× magnification. The second set has 201 images representing the normal epithelium of the oral cavity and 495 HIs of OSCC taken at 400× magnification. These images were obtained using a Leica ICC50 HD microscope from hematoxylin and eosin (H&E)-stained tissue slides, then collected, processed, and organized by medical professionals from 230 patients. Table 1 presents the resolution, type, and quantity of HIs images included in the oral tumor dataset, while Figure 3 provides examples from the dataset [42].

### 3.2. Experimental Methodology

To identify OSCC from HIs, we developed a DL model specifically designed to detect this type of tumor using the oral tumor dataset from the multi-cancer dataset. Our model employed the EfficientNet-B3 architecture, enhanced by the LRT technique, which was chosen for its remarkable accuracy in analyzing HIs.

**Figure 4:**
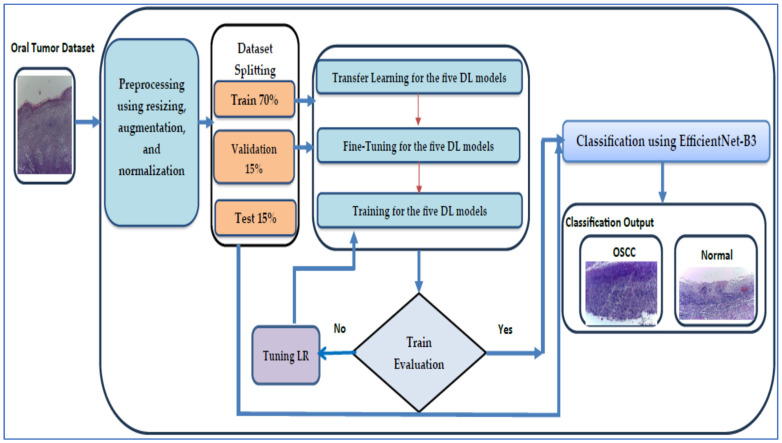
The proposed EfficientNet-B3 architecture.

We assessed the performance of the EfficientNet-B3 model with the oral tumor dataset. The structure of our proposed DL model is illustrated in Figure 4, while Algorithm 1 offers a comprehensive description of the processes involved in training and fine-tuning five DL models, namely EfficientNet-B1, EfficientNet-B2, EfficientNet-B3, DenseNet169, and InceptionV3. The steps for implementing the proposed DL model are outlined as follows:

**Phase 1 (oral tumor dataset preprocessing)**: In the initial phase, the oral tumor dataset was obtained from Kaggle and underwent preprocessing [40]. During the preprocessing stage, the HIs from the oral tumor dataset was augmented, re-scaled, and normalized.

**Phase 2 (oral tumor dataset splitting)**: In the second phase, the oral tumor dataset was divided into the following three parts: 70% for the training set containing 6998 HIs, 15% for the test set with 1501 HIs, and 15% for the validation set, also comprising 1501 HIs.

**Phase 3 (five pre-trained DL models)**: In the third step, we selected five advanced pre-trained DL models that were trained on the ImageNet dataset. These models included EfficientNet-B1, EfficientNet-B2, EfficientNet-B3, DenseNet169, and InceptionV3.

**Phase 4 (fine-tuning for the five DL models)**: In the fourth step, the EfficientNet-B1, EfficientNet-B2, EfficientNet-B3, DenseNet169, and InceptionV3 models were fine-tuned on the training set of the oral tumor dataset to achieve the optimal hyperparameters.

**Phase 5 (applying the LRT)**: Throughout the training process of the five DL models, we employed the LRT and assessed the error of the training set. If the error was deemed excessive, the model underwent re-training. If not, we proceeded to evaluate the error of the testing set.

**Phase 6 (binary classification with a constant LR)**: We carried out an experiment focused on binary classification with a constant LR using the oral tumor dataset. In addition, we evaluated the performance by using various metrics, including sensitivity, accuracy, precision, F1-score, and specificity.

**Phase 7 (binary classification)**: The models EfficientNet-B0, EfficientNet-B1, EfficientNet-B2, DenseNet169, and InceptionV3 utilizing the LRT were employed to execute a binary classification on the oral tumor dataset. Furthermore, the performances of the five DL approaches were evaluated using the assessed metrics.
**Algorithm 1:** Fine-tuning for the five DL models.1**Input** → Oral Tumor dataset as OD.2**Output** ← Five fine-tuned DL Models.3**BEGIN**4   **STEP 1**: **Preprocessing of HIs**5      *Apply* augmentation.6      **FOR EACH** HI **IN** the OD **DO**7             *Resize* HI to 512 × 512 pixels.8             *Normalize* HI from the range [0, 255] to [0, 1].9
   **STEP 2: Data Splitting**
10      **SPLIT** OD and **INTO**11         *Training set* →70%.12         *Testing set* →15%.13         *Validation set* →15%.14
   **STEP 3: Five DL Models Pre-Training**
15      **FOR EACH** DM IN [EfficientNet-B3, EfficientNet-B1, EfficientNet-B2, DenseNet169, and InceptionV3] **DO**16         *Load* DM. 17         *pre-trained* DM on the ImageNet dataset.18
      **END FOR**
19
   **STEP 4: Five DL Models Fine-Tuning**
20          **FOR EACH** DM IN [EfficientNet-B3, EfficientNet-B1, EfficientNet-B2, DenseNet169, and InceptionV3] **DO**21             *Fine-tune* DM on the training set of OD.22             **IF** error of training < Threshold **GOTO** Step 2523
             **ELSE**
24               **GOTO** Step 2025
             **END IF**
26
          **END FOR**
27
   **STEP 5: Five DL Models Evaluation**
28          **FOR EACH** DM IN [EfficientNet-B3, EfficientNet-B1, EfficientNet-B2, DenseNet169, and InceptionV3] **DO**29             *Evaluate* DM on the testing set of OD.30             **IF** error of testing < Threshold **GOTO** 3331
             **ELSE**
32               **GOTO** Step 20.33
             **END IF**
34                *Evaluate* DM using the assessed metrics.35
          **END FOR**


#### 3.2.1. Data Preprocessing

Data preprocessing plays a crucial role in any data analysis, DL, or ML workflow. This phase is dedicated to transforming raw data into a suitable format for effective analysis and modeling. Data preprocessing for HIs in DL consists of several critical steps aimed at enhancing model performance. The common sequence of preprocessing methods applied to HIs is as follows:

1. HI resizing ensures uniform dimensions across images for consistent input to the DL model. It adjusts all images to a specified resolution (for instance, 224 × 224 pixels for EfficientNet-B3) to meet input layer specifications.
2. HI normalization adjusts pixel intensity values to a standardized range, facilitating better model convergence. It scales pixel values to a range of [0, 1] by dividing by 255 (for RGB images) or standardize using the mean and standard deviation calculated from the dataset.
3. Data augmentation enhances data variability and minimizes the risk of overfitting through various transformations. Data augmentation is carried out using the following techniques:
  - Rotation: Randomly rotate images within a limited range (e.g., ±15 degrees).
  - Flipping: Implement horizontal or vertical flips.
  - Zooming: Randomly zoom in on images.
  - Shifting: Apply minor translations both horizontally and vertically.
  - Brightness/contrast adjustments: Change brightness or contrast to mimic different imaging conditions.

In our methodology, we utilized a variety of data augmentation techniques. We rotated the images up to 10 degrees, altered their width and height by a maximum of 10% of the total image size, and applied shearing and zooming changes of 10%. To increase variability, we incorporated random horizontal flips. Brightness levels were adjusted within a range of 0.2 to 1.2 to simulate different lighting conditions. Furthermore, the images were resized to 512 × 512 pixels, and the pixel values were normalized from the range of [0, 255] to [0, 1]. The number of images in the oral tumor dataset of the multi-cancer dataset, following these data augmentation processes, is 10,000 HIs (where 5000 images correspond to the OSCC class and 5000 images correspond to the normal class).

#### 3.2.2. The LRT

In optimal settings for LR, CNNs converge after several iterations. The LR determines the amount of loss that is backpropagated using GDA to move towards the minimum point. Once the GDA reaches this minimum, significant advancements occur, but they come at a high computational expense. Conversely, in the LRT dynamic method, the LR adjusts by a predetermined amount if there is no improvement in accuracy after a few epochs or if it reaches a minimal point. In contrast, the non-LRT method maintains a constant LR throughout training or gradually decreases it with each epoch. Our LRT approach is detailed in Algorithm 2.
**Algorithm 2:** The algorithm of the LRT1**Input** → no_epoch, fr, 1.0 > fr > 0.02**Output** ← LRT3**BEGIN**4
   **DO**
5
      **DO**
6             *READ* epoch.7             **IF** (epoch ≠0)8                no_epoch =epoch9
             **ELSE**
10
                **HALT**
11
             **END IF**
12
      **WHILE (no_epoch = 0)**
13   *READ* dwell_Val 14   **IF** dwell_Val *== True*
**THEN**15         *Compute* Cu_vld_loss16         *Compute* Cu_tn_accu17         **IF** Cu_vld_loss > P_vld_loss **THEN**18             Cu_W = P_W19             Cu_B = P_B20             N_LR = Cu_LR * fr21
         **END IF**
22         **IF** Cu_tn_ac < P_tn_ac **THEN**23             Cu_W = P_W24             Cu_B = P_B25             N_LR = Cu_LR * fr26
         **END IF**
27
   **END IF**
28   no_epoch = no_epoch − 129   **WHILE** (no_epoch = 0)30**END**

Algorithm 2 utilizes the LRT. When the current validation loss exceeds the prior validation loss (suggesting that the model may be overfitting or failing to converge effectively) or when the current training accuracy drops below the previous training accuracy, the weights from the last epoch are reinstated. The LR is then modified. The updated LR is determined by multiplying the existing LR by a factor that is less than 1.0 and greater than 0.0.

#### 3.2.3. EfficientNet Models

EfficientNet comprises a series of CNNs designed to enhance both the accuracy and efficiency of image classification tasks. It employs a technique known as compound scaling, which modifies the network’s depth, width, and resolution by using specific scaling coefficients. This innovative architecture strikes a balance between computational efficiency and model performance, surpassing many earlier CNN designs. By implementing this balanced scaling approach, EfficientNet models can achieve impressive accuracy with a reduced number of parameters, rendering them highly efficient across various computing platforms [44].

EfficientNet-B0 serves as the foundational model in the EfficientNet series, aimed at maximizing accuracy while minimizing computational demands. The development of this model utilized a neural architecture search (NAS) to uncover the most effective design for achieving a balance between efficiency and performance. A key feature of EfficientNet-B0 is its compound scaling method, which concurrently adjusts the model’s depth (number of layers), width (number of channels), and input resolution (size of the input image). This results in EfficientNet-B0 achieving notable accuracy while using fewer parameters than conventional architectures. It is specifically designed for an input resolution of 224 × 224 pixels. EfficientNet-B0 incorporates mobile inverted bottleneck convolution layers (MBConv) and integrates squeeze-and-excitation (SE) blocks. These SE blocks are crucial as they selectively enhance significant feature channels, improving the model’s ability to effectively represent and process data. With approximately 5.3 million parameters, EfficientNet-B0 offers excellent performance for its size, making it a preferred option for applications requiring lightweight models that do not compromise on performance, particularly in mobile and embedded contexts [44].

Building on the foundation laid by EfficientNet-B1, EfficientNet-B2 increases the depth, width, and resolution of input images through a balanced compound scaling approach. This technique proportionately adjusts these elements, leading to enhanced performance. With an input resolution of 260 × 260 pixels, EfficientNet-B2 surpasses B1 that has input resolution 240 × 240 slightly, enabling it to capture more complex features. The expanded width and depth of EfficientNet-B2 compared to B1 allows it to learn advanced feature representations while maintaining computational efficiency. Similar to its EfficientNet counterparts, B2 utilizes MBConv layers in conjunction with SE blocks, which enhance the model’s focus on critical aspects within an image. As a result, EfficientNet-B2 achieves greater accuracy than B1, although it requires more computational resources, making it particularly effective in scenarios where performance and efficiency are crucial [44].

EfficientNet-B3 employs a compound scaling strategy that boosts network performance by increasing its width, depth, and input resolution beyond the base model, EfficientNet-B0. This method applies to fixed coefficients to ensure balanced growth across all dimensions. Consequently, it significantly enhances the network’s ability to identify intricate image patterns while efficiently managing computational costs [44].

**Figure 5:**
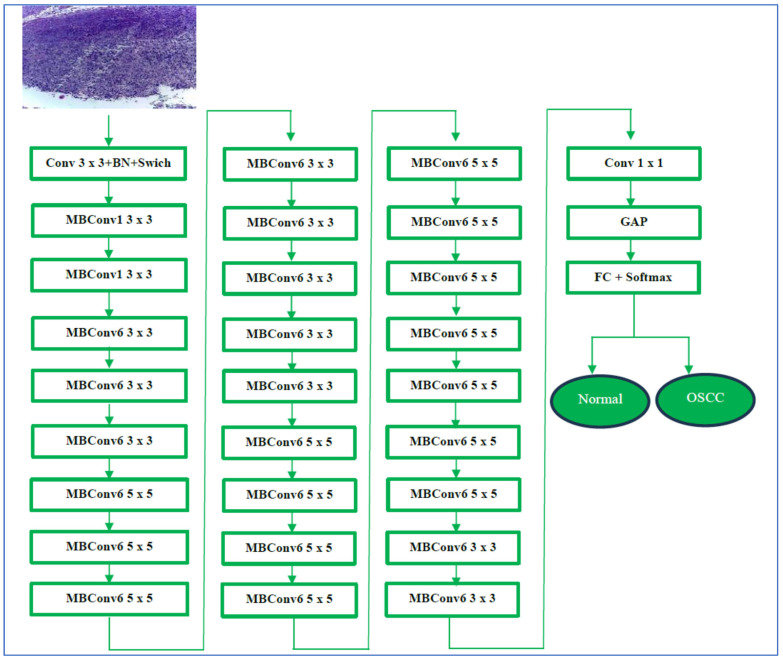
The EfficientNet-B3 architecture.

EfficientNet-B3 is designed to work with an input resolution of 300 × 300 pixels. Its network width is approximately 1.2 times that of EfficientNet-B0, and its depth is about 1.4 times greater. This dimensional increase enables EfficientNet-B3 to utilize a larger number of channels and layers, enhancing its capability to learn complex and detailed features from images. Additionally, EfficientNet-B3 incorporates MBConv and SE optimization techniques, which help emphasize important features. Despite these improvements, it remains efficient, requiring fewer parameters than many traditional architectures. This efficiency renders it suitable for a variety of applications, including those on devices with limited resources, such as mobile phones. In summary, EfficientNet-B3 provides a significant accuracy enhancement over EfficientNet-B0, while its efficient design supports scalability and high performance [44]. EfficientNet-B3’s architecture is depicted in Figure 5.

#### 3.2.4. DenseNet169

**Figure 6:**
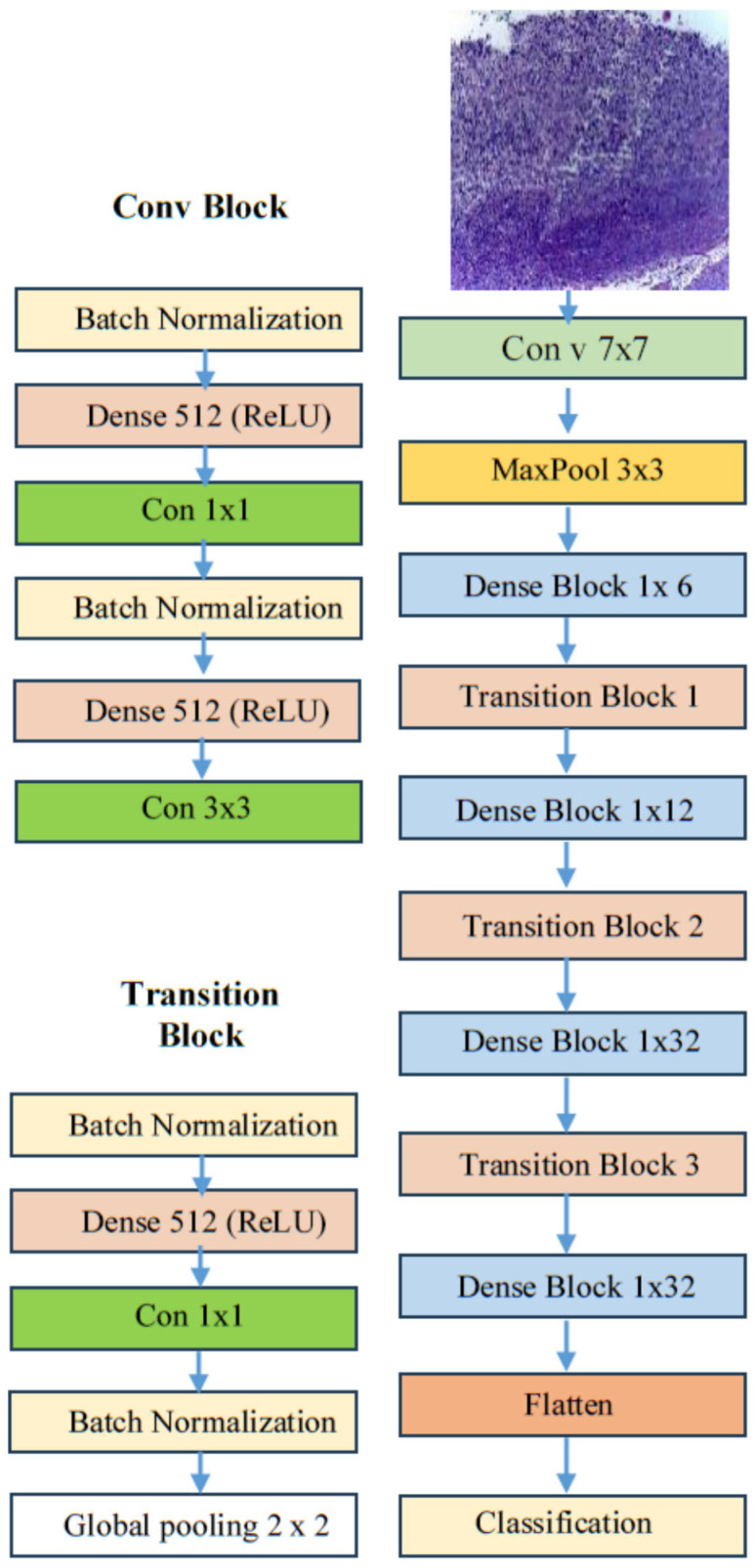
The DenseNet169 architecture.

DenseNet169, which stands for densely connected convolutional network with 169 layers, is a DL architecture introduced by Gao Huang, Zhuang Liu, Laurens van der Maaten, and Kilian Q. Weinberger in their 2017 paper titled “Densely Connected Convolutional Networks.” [45]. The DenseNet architecture, as depicted in Figure 6, was specifically designed to tackle the vanishing gradient problem and to improve feature propagation in very deep networks. These issues were prevalent in earlier architectures, such as VGG and ResNet [45].

In contrast to traditional convolutional networks, where each layer is only connected to the following layer, DenseNet employs a method known as dense connectivity. In this structure, each layer is connected to every other layer in a feed-forward manner. Consequently, the input for any given layer includes the feature maps from all preceding layers, and the feature maps generated by that layer are forwarded to all subsequent layers [45]. This dense connectivity facilitates the following:

- Feature reuse: Enhances the use of features learned by earlier layers.
- Reduced parameters: Decreases the number of parameters needed, which can improve efficiency.
- Enhanced gradient flow: Improves the flow of gradients during training, making the network more efficient and easier to train.

These characteristics make DenseNet169 a significant advancement in DL architecture [45]. The DenseNet169 architecture is composed of 169 layers, which include dense blocks and transition layers, as follows [45]:

- Dense blocks: These are sequences of layers where each layer performs a convolution operation, followed by batch normalization and a non-linear activation function, typically ReLU.
- Transition layers: Located between dense blocks, these layers reduce the spatial dimensions of the feature maps through pooling and adjust the number of channels using 1 × 1 convolutions.

This design allows the network to be computationally efficient while still maintaining a high level of representational power.

One of the main advantages of DenseNet169 is its parameter efficiency. By reusing features from all previous layers, it requires fewer parameters compared to other architectures of similar depth, such as ResNet, while achieving competitive or even superior performance across various tasks. Additionally, the dense connectivity helps address the vanishing gradient problem, making it possible to train very deep networks without significant performance loss [45].

DenseNet169 is widely used in computer vision tasks, such as image classification, object detection, and segmentation. Its capacity to capture complex patterns and hierarchical features makes it especially effective in medical imaging, like identifying tumors in MRI or CT scans, as well as in natural image analysis, such as classifying objects in the ImageNet dataset. The architecture’s efficiency and robustness also make it a popular choice for environments with limited resources, including mobile and embedded systems where computational power is constrained. Overall, DenseNet169 represents a major advancement in deep learning, combining innovative architectural design with practical advantages, which has established it as a key component of modern convolutional neural networks [45].

#### 3.2.5. InceptionV3

**Figure 7:**
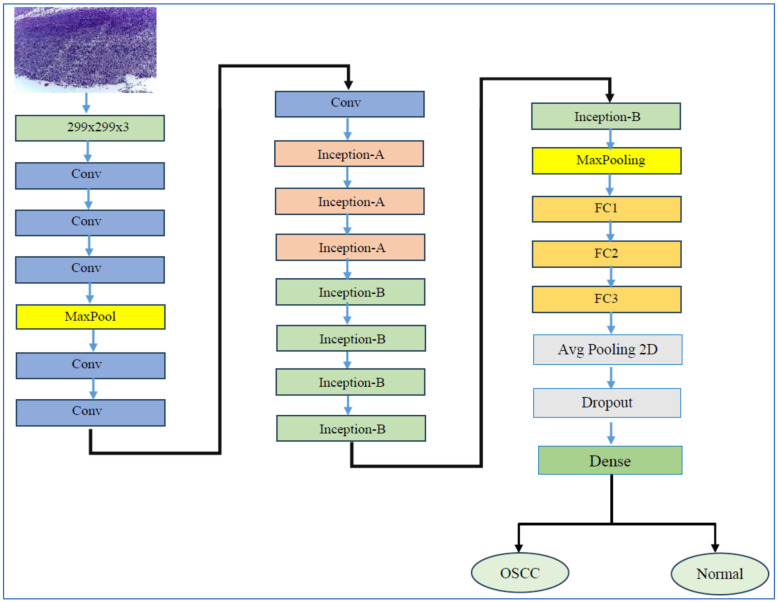
InceptionV3’s architecture.

InceptionV3 is a CNN architecture that marks a significant advancement in the development of DL models for computer vision tasks. InceptionV3’s architecture is depicted in Figure 7. Introduced by researchers at Google in 2015, it builds upon its predecessors, namely InceptionV1 (GoogLeNet) and InceptionV2 [46]. The family of Inception architectures were specifically designed to tackle the challenges of computational efficiency and accuracy in image classification. InceptionV3 notably enhanced the idea of the “Inception module”, which utilizes multiple filter sizes (such as 1 × 1, 3 × 3, and 5 × 5) within the same layer. This design enables the model to capture features at various scales, facilitating the efficient extraction of both fine-grained and coarse-grained features from images. Furthermore, InceptionV3 introduced several key innovations, including the factorization of convolutions—replacing a 5 × 5 convolution with two 3 × 3 convolutions—and the implementation of batch normalization. These improvements help stabilize the training process and enhance convergence. As a result, InceptionV3 not only became more computationally efficient but also achieved state-of-the-art performance on benchmark datasets, such as ImageNet [46].

One of the main benefits of InceptionV3 is its effectiveness in balancing accuracy with computational cost. The model employs factorized convolutions and auxiliary classifiers, which help to lower the number of parameters and computational operations compared to earlier models, like VGGNet, while still achieving high accuracy. This efficiency makes InceptionV3 a great choice for use in environments with limited resources, such as mobile devices or embedded systems. Additionally, the modular design of InceptionV3 allows it to be easily adapted for various tasks beyond just image classification. It can be applied in such areas as object detection, segmentation, and transfer learning for specific applications. For example, InceptionV3 has been extensively utilized in medical imaging for various tasks, such as tumor detection and diabetic retinopathy classification. It is also used in natural language processing, particularly for visual question answering systems [46].

## 4. Experimental Results and Insights

### 4.1. Measured Performance Metrics

The effectiveness of the five DL models—EfficientNet-B3, EfficientNet-B1, EfficientNet-B2, DenseNet169, and InceptionV3—is evaluated using Equations (1)–(7), as follows:

(1) $$\mathrm{Accuracy}=\frac{\left(TP+TN\right)}{\left(TP+FP+TN+FN\right)}$$

(2) $$\mathrm{Precision}=\frac{TP}{\left(TP+FP\right)}$$

(3) $$\mathrm{Sensitivity}=\frac{TP}{\left(TP+FN\right)}$$

(4) $$\mathrm{Specificity}=\frac{TN}{(TN+FP}$$

(5) $$F1-\mathrm{score}=\frac{2\times \mathrm{Precision}\;\times \mathrm{Recall}}{\mathrm{Precision}+\mathrm{Recall}}$$

(6) $$\mathrm{False}\;\mathrm{Negative}\;\mathrm{Rate}\;\left(\mathrm{FNR}\right)=\frac{FN}{TP+FN}$$

(7) $$\mathrm{Negative}\;\mathrm{Predictive}\;\mathrm{Value}\;\left(\mathrm{NPV}\right)=\frac{TN}{TN+FN}$$

where true positive (*TP*) means that the model correctly predicts a case as positive when it actually is positive (e.g., it correctly detects a tumor), true negative (*TN*) means that the model correctly predicts a case as negative when it truly is negative (e.g., no tumor is present, and the model predicts that correctly), false positive (*FP*) is when the model wrongly predicts a case as positive, but it is actually negative (e.g., it says that there is a tumor when there is not), and false negative (*FN*) is when the model wrongly predicts a case as negative when it is actually positive (e.g., it misses a tumor that is present). Accuracy shows how often the model is correct overall—it is the ratio of correct predictions to total predictions. Precision (or positive predictive value) shows how many of the predicted positive cases were actually positive—it reveals how reliable the positive predictions are. Sensitivity (or recall) measures how well the model finds all the actual positive cases—it is important when missing a positive case is critical. Specificity indicates how well the model correctly identifies negative cases, which is useful when it is important to avoid false alarms. F1-score is the balance between precision and sensitivity and is especially helpful when classes are imbalanced. False negative rate (FNR) shows the proportion of actual positive cases the model missed—it is the opposite of sensitivity. Negative predictive value (NPV) shows how many of the predicted negative cases are truly negative, indicating how reliable the negative predictions are.

### 4.2. Experiments Setup

In this study, we performed experiments using Python 3 on the Kaggle platform, on a laptop equipped with an AMD Ryzen™ 7 7735HS processor, 8 GB of RAM, and a 512 GB SSD, operating on Windows 11. The hyperparameters employed in this experiment, along with their corresponding values, are detailed in Table 2.

By selecting a batch size of 115, this value was empirically determined based on hardware constraints and performance considerations. In addition, we employed early stopping based on validation loss, and, in most cases, performance plateaued before 30 epochs.

### 4.3. Performance Assessment of the Proposed Model

The multi-cancer dataset includes the oral tumor dataset. The oral tumor dataset was divided as follows: 70% was designated for the training set (6998 HIs), 15% was designated for the test set (1501 HIs), and 15% was designated for the validation set (1501 HIs). We conducted two experiments which focused on applying transfer learning by pre-training five DL models. In the first experiment, we employed the LRT for our training process. The second experiment involved the implementation of a constant LR algorithm. In both experiments, we engaged in the transfer learning process.

The transfer learning process has two stages. In stage one, we initiated supervised pre-training of various DL models, specifically EfficientNet-B3, EfficientNet-B1, EfficientNet-B2, DenseNet169, and InceptionV3, using the ImageNet dataset. In stage two, we refined these models further by utilizing the training set from the oral tumor dataset. After completing each experiment, we evaluated the performance of the five DL models by applying metrics based on Equations (1)–(7).

**Figure 8:**
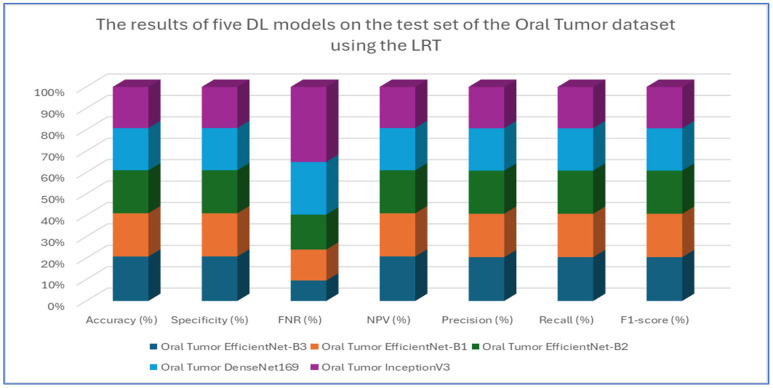
The results of five DL models on the test set of the oral tumor dataset using the LRT.

The results from the first experiment involving EfficientNet-B3, EfficientNet-B1, EfficientNet-B2, DenseNet169, and InceptionV3 on the oral tumor test set are summarized in Table 3 and Figure 8. Among the models evaluated, EfficientNet-B3 achieved the highest performance metrics on the oral tumor dataset, with an accuracy of 99.84%, specificity and NPV of 99.92%, and a recall and F1-score of 97.93%. Its precision was notably high at 97.94%, while the FNR was minimal at 2.07%, establishing it as the leading model for this dataset. The high recall and low FNR indicate EfficientNet-B3’s effectiveness in identifying TP cases, thus reducing the likelihood of missed detections. This low FNR makes EfficientNet-B3 particularly suitable for clinical applications, where failing to detect a tumor can have serious implications.

EfficientNet-B1 showed slightly better performance than EfficientNet-B2, with an accuracy of 96.87% compared to 96.47%, and a lower FNR of 3.13% versus 3.53%. This suggests that the increased capacity of EfficientNet-B3, achieved through compound scaling in depth, width, and resolution, likely played a significant role in its superior performance.

In contrast, DenseNet169 and InceptionV3 performed less effectively than the EfficientNet variants, with DenseNet169 achieving an accuracy of 94.67% and InceptionV3 recording the lowest accuracy at 92.41%. The higher FNR of InceptionV3 at 7.59% points to its challenges in capturing essential tumor features compared to the more advanced architectures. The consistent drop in performance from EfficientNet-B3 to InceptionV3 highlights the importance of architectural improvements over time. For example, EfficientNet’s implementation of compound scaling and optimized convolutional blocks has likely improved feature extraction efficiency, while InceptionV3’s reliance on multi-branch inception modules, though innovative at its inception, may not have been as effective for the complexities of the oral tumor dataset.

In conclusion, this analysis underscores the superiority of EfficientNet-B3 in oral tumor detection, while older models, like InceptionV3, faced challenges in addressing contemporary medical imaging tasks. The findings emphasize the significance of advancing neural network designs that prioritize both accuracy and computational efficiency for real-world applications.

**Figure 9:**
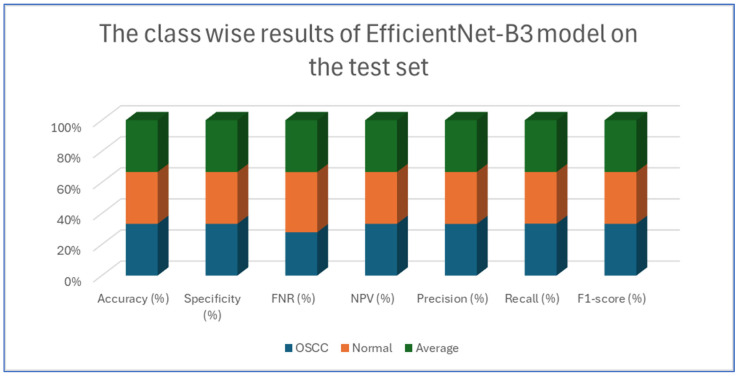
The class-wise results of EfficientNet-B3 on the test set of the oral tumor dataset using the LRT.

Table 4, Table 5, Table 6, Table 7 and Table 8 presents class-wise evaluation metrics for the five DL models on the test set of the oral tumor dataset that contains two classes, namely OSCC and normal. The evaluation results shown in Table 4 and Figure 9 illustrate the performance of the EfficientNet-B3 model in distinguishing between OSCC and normal cases from the dataset. For the OSCC class, the model achieved an accuracy of 99.84%, a specificity of 99.90%, and an FNR of 1.73%. The NPV was 99.93%, showcasing the model’s ability to accurately predict the absence of OSCC, while its precision, recall, and F1-score were 97.62%, 98.27%, and 97.94%, respectively. For the normal class, the model achieved the same accuracy of 99.84%, a specificity of 99.93%, and an FNR of 2.40%. The NPV was 99.90%, with precision, recall, and F1-score recorded at 98.26%, 97.60%, and 97.93%, respectively.

On average across both classes, the model demonstrated a 99.84% accuracy, a specificity of 99.92%, an FNR of 2.07%, an NPV of 99.92%, and both precision and recall scores as well as F1-score at 97.93%. These results highlight the model’s strong capability to reliably detect both OSCC and normal tissues, with minimal FNs, making it a valuable tool for diagnostic applications.

**Figure 10:**
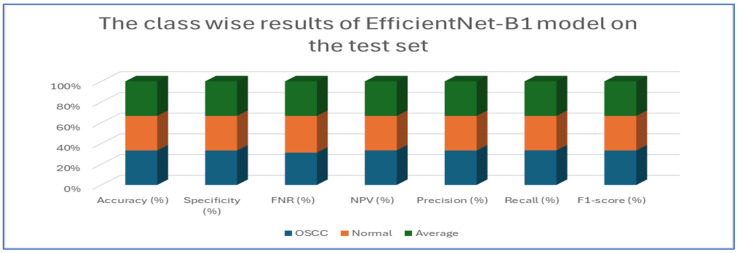
The class-wise results of EfficientNet-B1 on the test set of the oral tumor dataset using the LRT.

Table 5 and Figure 10 present the performance of the EfficientNet-B1 model when applied to two classes, namely OSCC and normal. For the OSCC class, the model achieved an accuracy of 96.87%, a specificity of 96.67%, and an FNR of 2.93%. The NPV was recorded at 97.05%, with a precision of 96.68%, a recall of 97.07%, and an F1-score of 96.88%. In the case of the normal class, the model maintained the same overall accuracy of 96.87% but demonstrated a slightly higher specificity at 97.07% and an FNR of 3.33%. The NPV for this class was 96.68%, while the precision was 97.05%, the recall was 96.67%, and the F1-score was 96.86%. The average performance across both classes was summarized with values of 96.87% for accuracy, specificity, precision, recall, and F1-score, while the average FNR was 3.13% and the average NPV was 96.87%.

Overall, the analysis indicated that the EfficientNet-B1 model exhibited consistent and balanced performance across the OSCC and normal classes, maintaining high accuracy and robust performance metrics throughout.

**Figure 11:**
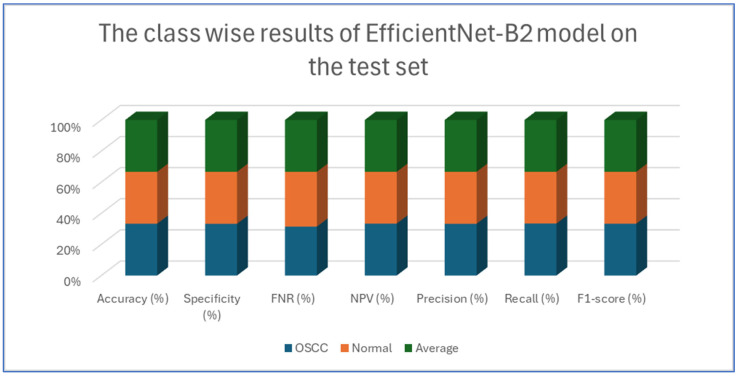
The class-wise results of EfficientNet-B2 model on the test set of the dataset using the LRT.

Table 6 and Figure 11 provide an evaluation of the EfficientNet-B2 model across two classes—OSCC and normal—as well as their averaged metrics. The analysis showed that for the OSCC class, the model achieved an accuracy of 96.47%, with a specificity of 96.27% and an FNR of 3.33%. In addition, the NPV was 96.65%, the precision was 96.29%, the recall reached 96.67%, and the F1-score was 96.48%. For the normal class, the model maintained the same overall accuracy of 96.47%, while the specificity improved slightly to 96.67%. However, the FNR increased slightly to 3.73%. The NPV for this class was 96.29%, and the precision and recall were 96.65% and 96.27%, respectively, which resulted in an F1-score of 96.46%.

This analysis demonstrates that the model performed robustly and consistently across both categories, maintaining high accuracy and balanced performance metrics.

**Figure 12:**
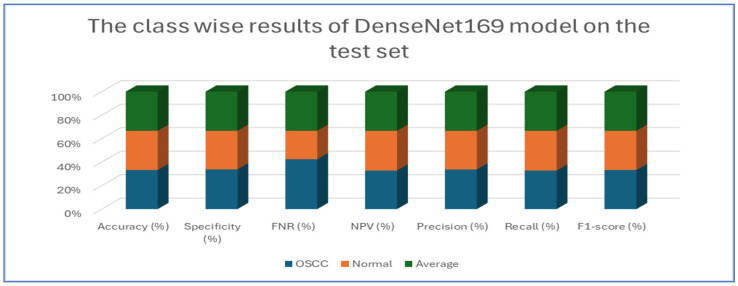
The class-wise results of DenseNet169 on the test set of the oral tumor dataset using the LRT.

Table 7 and Figure 12 present the performance metrics for the DenseNet169 model applied to a dataset containing OSCC (oral squamous cell carcinoma) and normal samples. For the OSCC class, the model achieved an accuracy of 94.67%. It exhibited a high specificity of 96.13% and maintained a low FNR of 6.79%. The NPV was calculated at 93.39%, while precision reached 96.02%. The recall was slightly lower at 93.21%, resulting in an F1-score of 94.59%. In the case of the normal class, the model also achieved an accuracy of 94.67%. However, the specificity for this class was lower at 93.21%, and the FNR was reduced to 3.87%. The NPV improved to 96.02%, and the precision was noted to be 93.39%. Conversely, the recall increased to 96.13%, leading to a slightly higher F1-score of 94.74%.

Overall, the analysis indicates that the DenseNet169 model performed consistently across both classes. It demonstrated a strong balance between recall and precision, as evidenced by the similar F1-scores. These results suggest that the model effectively distinguished between OSCC and normal cases, showcasing robust and reliable diagnostic capabilities.

**Figure 13:**
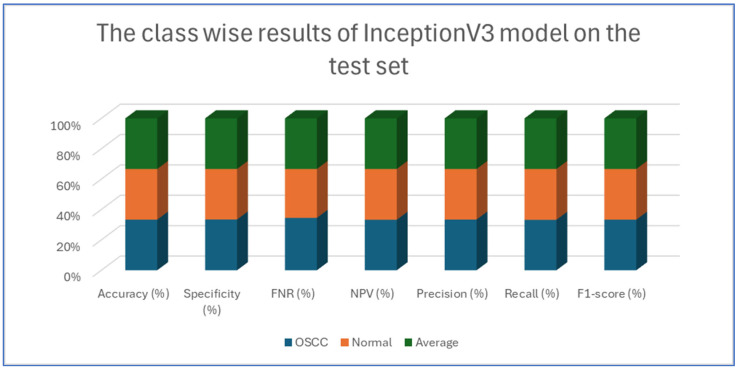
The class-wise results of InceptionV3 on the test set of the oral tumor dataset using the LRT.

Table 8 and Figure 13 present an analysis of the InceptionV3 model’s performance on a dataset containing two classes, namely OSCC and normal. For the OSCC class, the model achieved an accuracy of 92.41%, a specificity of 92.67%, an FNR of 7.86%, and an NPV of 92.18%. The precision was 92.64%, the recall was 92.14%, and the F1-score was 92.39%. For **the normal class, the model** also reached an accuracy of 92.41%, but the specificity was slightly lower at 92.14%, while the FNR was 7.33% and the NPV was 92.64%. The precision and recall for the normal class were 92.18% and 92.67%, respectively, and the F1-score was 92.42%.

The analysis indicates that the InceptionV3 model performed consistently well across both classes, with only minor variations between the OSCC and normal classes. The balanced metrics suggest that the model achieved reliable performance on the dataset.

**Figure 14:**
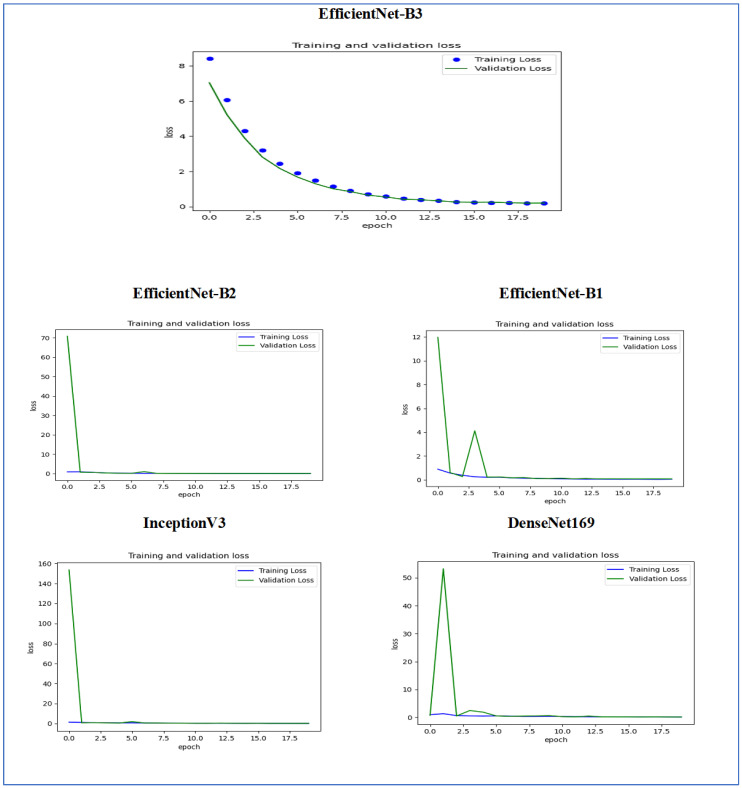
The training and validation loss for the five DL models tested on the oral tumor dataset using the LRT.

Figure 14 illustrates the training and validation loss of the five DL models when evaluated on the test set of the oral tumor dataset through the LRT. For EfficientNet-B3, the training loss, represented by the blue dots, started at a high point of approximately 9 and rapidly decreased during the initial epochs, showcasing the model’s quick learning capability. Similarly, the validation loss, shown by the green line, also experienced a significant initial decline, mirroring the trend of the training loss. As the training progressed over the epochs, both training and validation losses stabilized and approached low values near zero. This convergence suggested that the model achieved a steady state with minimal error. Additionally, the close correspondence between the training and validation losses in the later epochs indicated that overfitting was not a major issue for this model.

For EfficientNet-B1, the training loss began at a higher value and demonstrated a steady decline as the number of epochs increased. This trend indicates that the model was effectively learning and enhancing its performance on the training data. In contrast, the validation loss also started higher but displayed a more variable pattern compared to the training loss. This fluctuation suggests that although the model was generalized to some degree, there were instances where it had difficulty with the validation data, potentially indicating overfitting or the need for further tuning. By the conclusion of 17.5 epochs, both the training and validation losses had decreased. However, the validation loss was still slightly higher than the training loss, which is a common occurrence in many machine learning scenarios. Overall, while the model made progress in reducing loss, there is likely room for additional optimization to achieve improved generalization.

For EfficientNet-B2, the training loss consistently decreased over the epochs, indicating that the model was effectively learning from the training data. The validation loss also showed a decline over time but displayed more fluctuations compared to the training loss. This variability suggests that the model’s performance on the validation data was less stable, which could indicate potential issues, such as overfitting or a need for further tuning of hyperparameters. By the final epoch, both the training and validation losses had decreased; however, the validation loss remained higher than the training loss. This is a common observation in model training. Overall, the trend shows that the model improved its performance, but there may be further opportunities to enhance its generalization capabilities.

For DenseNet169, the training loss started at a higher value and showed a steady decline throughout the epochs, indicating that the model was effectively learning and improving its performance on the training data. In contrast, the validation loss also began high but displayed more fluctuations compared to the training loss. These fluctuations suggest that the model’s performance on the validation data was less stable, which could indicate potential overfitting or the need for further optimization. By the end of the 17.5 epochs, both the training and validation losses had decreased. However, the validation loss remained higher than the training loss, which is a common occurrence in many training scenarios. Overall, the model showed progress in reducing loss, but there may have been opportunities to enhance its generalization on the validation data.

For InceptionV3, the training loss initially started at a higher value and showed a steady downward trend as the number of epochs increased. This indicates that the model was successfully learning and enhancing its performance on the training data. The validation loss also began at a higher value but displayed greater variability compared to the training loss. This variability suggests that the model’s performance on the validation data was less consistent, which could indicate issues, such as overfitting or the need for additional tuning. By the conclusion of 17.5 epochs, both the training and validation losses had decreased. However, it is important to note that the validation loss remained higher than the training loss, a common occurrence in many machine learning scenarios. Overall, the model showed progress in reducing loss, but there may still be opportunities for further optimization to improve generalization on the validation data.

**Figure 15:**
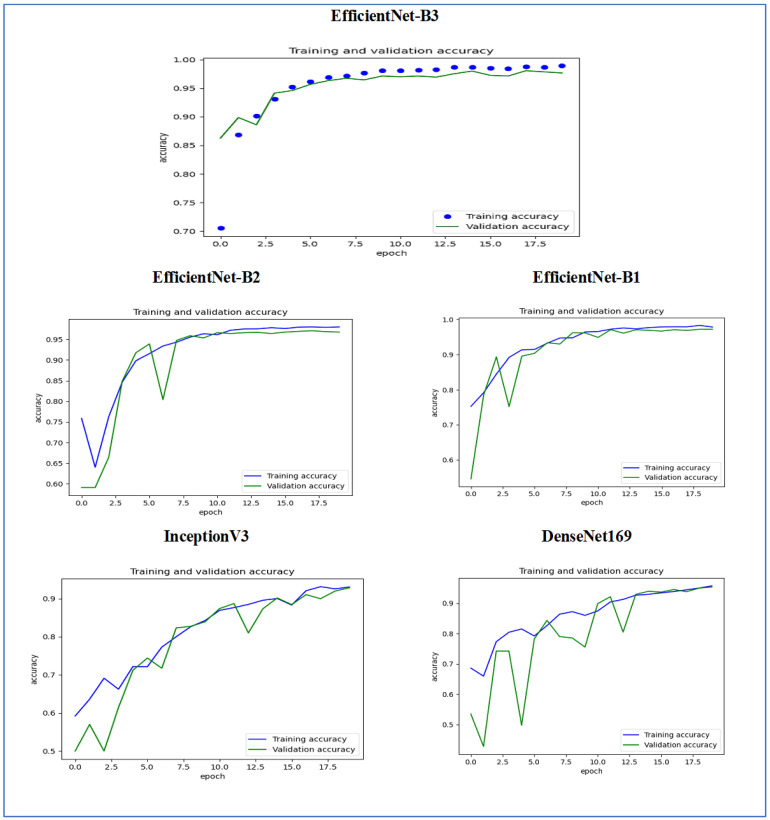
The training and validation accuracy for the five DL models tested on the oral tumor dataset using the LRT.

Figure 15 illustrates the training and validation accuracy of the five DL models when evaluated on the test set of the oral tumor dataset through the LRT. For EfficientNet-B3, the training accuracy increased rapidly in the early epochs and eventually plateaued at around 1.0. This indicates that the model successfully acquired knowledge from the training data. In a similar fashion, the validation accuracy showed an initial upward trend but stabilized at a slightly lower level than the training accuracy. Despite experiencing some minor variations, the general trend demonstrated a steady increase. Thus, the model was effectively generalizing to new, unseen data.

For EfficientNet-B1, the training accuracy consistently increased with each epoch, demonstrating that the model was effectively learning and enhancing its performance on the training dataset. The validation accuracy also rose over time; however, it showed some fluctuations, indicating that the model’s performance on the validation dataset was not as stable as on the training dataset. These fluctuations may suggest possible overfitting or the necessity for additional hyperparameter tuning. By the conclusion of 17.5 epochs, both training and validation accuracies had improved. Nonetheless, the validation accuracy remained slightly lower than the training accuracy, which is a common occurrence in many training scenarios. Overall, the model exhibited progress in accuracy improvements, but there may have been opportunities to further enhance its generalization on the validation dataset.

For EfficientNet-B2, the training accuracy consistently increased as the epochs progressed, indicating effective learning and improvement in performance on the training data. The validation accuracy also showed an upward trend, but with some fluctuations, suggesting that the model’s performance on the validation data was less stable compared to the training data. These fluctuations may indicate potential overfitting or a need for further hyperparameter tuning. By the end of 17.5 epochs, both the training and validation accuracies had improved, but the validation accuracy remained slightly lower than the training accuracy, which is common in many training scenarios. Overall, the model demonstrated progress in enhancing accuracy, but there may have been opportunities to further improve its generalization on the validation data.

For DenseNet169, the training accuracy started at a lower level and showed a consistent increase, rising from approximately 0.5 to 0.9 by the final epoch. This steady improvement indicated that the model was effectively learning patterns from the training data. The validation accuracy also increased overall but exhibited noticeable fluctuations during certain epochs, particularly between 5.0 and 12.5 epochs. These variations suggested instability in the model’s ability to generalize to unseen data, potentially due to overfitting or inconsistencies within the validation dataset. Despite these fluctuations, the validation accuracy reached a higher value by the end of the training period, although it remained slightly lower than the training accuracy. By the end of 17.5 epochs, the gap between training and validation accuracy had narrowed but still persisted, which is a common sign of mild overfitting. The model demonstrated strong learning capabilities on the training data, but it may have benefited from additional regularization techniques or early stopping to improve validation performance. Overall, the observed trends reflect successful learning while also highlighting opportunities for further optimization to enhance generalization.

For InceptionV3, the training accuracy began at approximately 0.5 and increased steadily, reaching a peak of 0.9 by the final epoch. This upward trajectory indicated that the model learned effectively from the training data, refining its predictions over time. The validation accuracy also improved overall, starting at a similar initial value but exhibiting fluctuations during intermediate epochs (e.g., between 5.0 and 12.5 epochs). These oscillations suggested variability in the model’s generalization performance, potentially due to overfitting or inconsistencies in the validation dataset. Despite these variations, the validation accuracy trended upward, ending close to training accuracy but slightly lagging behind. By the final epoch (17.5), the gap between training and validation accuracy had narrowed, though a small discrepancy persisted. This pattern aligned with typical training dynamics, where the model prioritizes fitting the training data but may struggle to generalize perfectly to unseen examples. The results implied that while the model achieved strong performance, additional regularization (e.g., dropout or weight decay) or early stopping might have further stabilized validation accuracy. Overall, the training process succeeded in improving model performance, but opportunities remained to enhance robustness on validation data.

**Figure 16:**
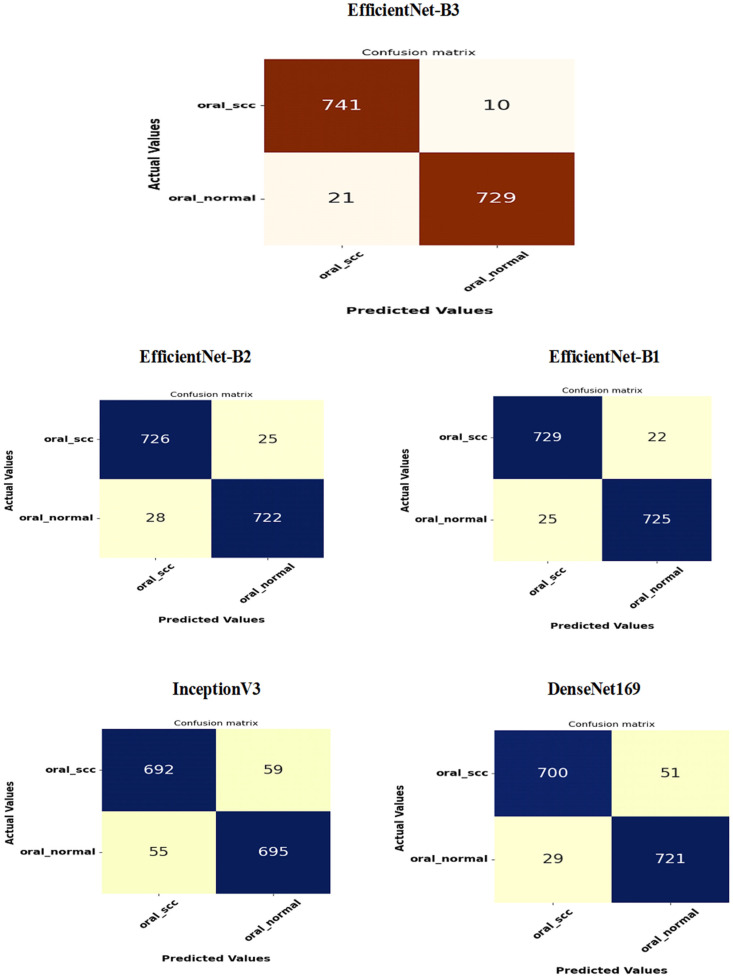
The confusion matrices for the five DL models tested on the oral tumor dataset using the LRT.

Figure 16 presents the confusion matrices for the five DL models, which were assessed across the test set of the oral tumor dataset utilizing the LRT. For EfficientNet-B3, the test set consists of 1501 HIs. The class distribution includes 750 HIs labeled as normal and 751 HIs labeled as OSCC. The EfficientNet-B3 model exhibited high accuracy, with the majority of its predictions being correct. It showed exceptional performance for the OSCC class, achieving an accuracy of 98.6%, with nearly all instances accurately classified. The model also performed well for the normal class, resulting in a few misclassifications and an accuracy of 97.2%. Overall, the EfficientNet-B3 model displayed strong capabilities in classifying oral cancer images into the OSCC and normal categories.

The EfficientNet-B1 model successfully classified 729 instances of the SCC class and 725 instances of the normal class. This resulted in an impressive overall performance, achieving an accuracy of 96.87%, along with balanced precision and recall for the SCC class. However, the model misclassified 22 SCC samples as normal and 25 normal samples as SCC. In a clinical context, the 22 missed SCC cases (FNs) could potentially delay critical treatment, while the 25 FPs might lead to unnecessary interventions. Overall, the model exhibited high accuracy with a relatively low number of misclassifications, demonstrating strong predictive capability for the task at hand.

The EfficientNet-B2 model demonstrated impressive overall performance with an accuracy of 96.47%. It achieved a well-balanced precision and recall for the SCC class. However, it misclassified 25 FNs, where SCC cases were incorrectly identified as normal. Additionally, there were 28 FPs, where normal cases were misclassified as SCC. In a clinical setting, the 25 missed SCC cases (FNs) could result in delays in critical treatment, while the 28 FPs could lead to unnecessary medical interventions.

The DenseNet169 model exhibited strong overall performance with an accuracy of 94.67%. However, it showed a slightly lower recall for the SCC class when compared to its precision. Specifically, the model recorded 51 FNs, where the SCC cases were incorrectly classified as normal, and 29 FPs, where the normal cases were misclassified as SCC. In a clinical setting, the 51 missed SCC cases (FNs) could potentially delay essential treatment, while the 29 FPs could result in unnecessary medical interventions. While the model demonstrated reasonable generalization capabilities, it had a higher rate of FNs than previous models.

The InceptionV3 model achieved a moderate overall performance with an accuracy of 92.40%. It exhibited balanced precision and recall for the SCC class. However, the analysis revealed 59 FNs, where SCC cases were incorrectly classified as normal, and 55 FPs, where normal cases were misclassified as SCC. In a clinical setting, the 59 missed SCC cases (FNs) could result in delays in critical treatment, while the 55 false alarms (FPs) could lead to unnecessary interventions. Overall, the model demonstrated acceptable generalization; however, it exhibited a higher rate of FNs and FPs compared to previous models.

**Figure 17:**
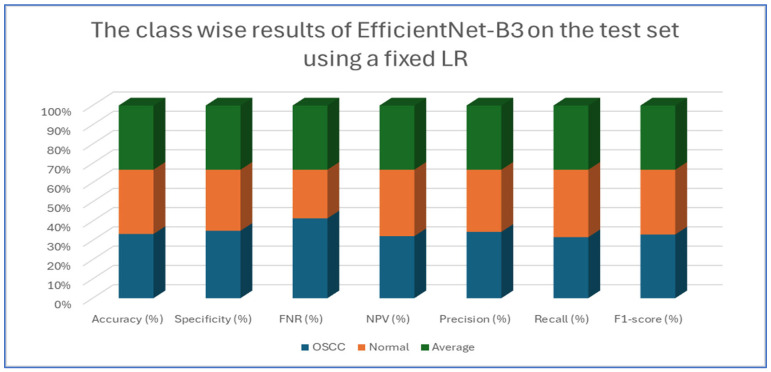
The class-wise results of EfficientNet-B3 on the test set of the oral tumor dataset using a fixed LR.

The results from the second experiment, which implemented a constant LR using EfficientNet-B3 on the oral tumor test set, are summarized in Table 9 and Figure 17. The EfficientNet-B3 model achieved an overall accuracy of 83.08%. For the OSCC class, the model demonstrated a specificity of 87.20% and an FNR of 21.04%. The NPV was recorded at 80.54%, while precision and recall were 86.07% and 78.96%, respectively. The F1-score for this class reached 82.36%.

In the normal class, the model attained a specificity of 78.96% and an FNR of 12.80%. The NPV was 86.07%, with precision and recall at 80.54% and 87.20%, respectively. The F1-score for this class was slightly higher at 83.74%.

On average, the model exhibited balanced performance across all metrics, with an overall accuracy, specificity, and recall of 83.08%, an FNR of 16.92%, and both NPV and precision at 83.30%. The final F1-score for the model was 83.05%, indicating consistent predictive capability across both classes.

A comparative analysis of the EfficientNet-B3 model performance using the LRT and a fixed LR was presented in Table 10.

**Figure 18:**
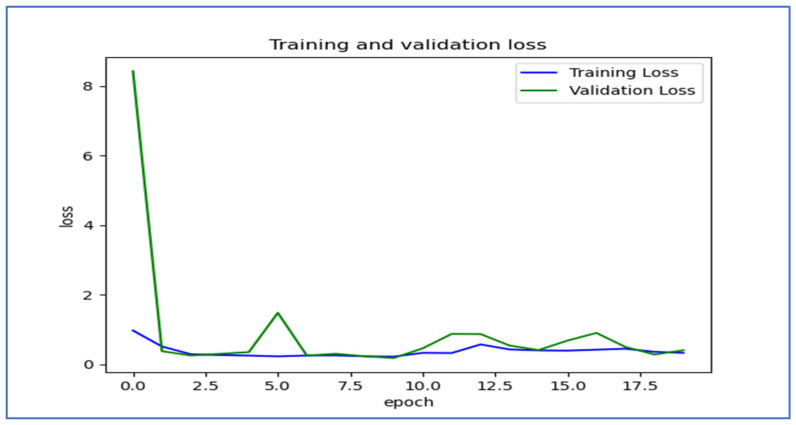
The training and validation loss for the EfficientNet-B3 model tested on the oral tumor dataset using a fixed LR.

Figure 18 shows the trends of training and validation loss over several epochs for the EfficientNet-B3 model. At the beginning, the validation loss was very high, indicating that the model made significant errors in its early stages. However, it decreased rapidly within the first few epochs, demonstrating that the model quickly learned important patterns in the data. Throughout the remaining epochs, both training and validation losses stayed low, though there were occasional fluctuations in the validation loss, which suggested some instability in generalization. Notably, around the 5th and 12th epochs, there were spikes in the validation loss, indicating potential overfitting or sensitivity to specific batches of validation data. By the later epochs, both losses converged to relatively stable values, showing that the model reached a balanced state with minimal error. Overall, the model displayed effective learning behavior, despite the spikes in validation loss.

**Figure 19:**
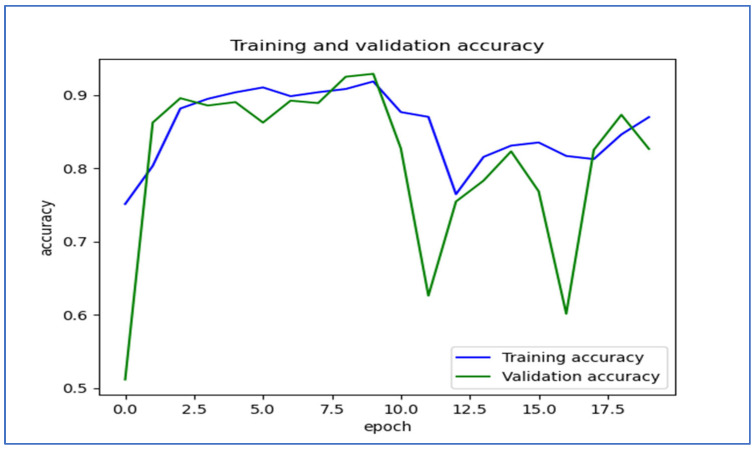
The training and validation accuracy for the EfficientNet-B3 model tested on the oral tumor dataset using a fixed LR.

Figure 19 shows the trends in training and validation accuracy over multiple epochs for the EfficientNet-B3 model. At first, both training and validation accuracy increased quickly, indicating effective learning by the model. Around the fifth epoch, the validation accuracy showed slight fluctuations, while the training accuracy remained stable and high. After about the 10th epoch, there were significant drops in validation accuracy, which suggested potential overfitting; the model performed well on the training set but had difficulty generalizing new data. Despite these fluctuations, both accuracies recovered somewhat, with training accuracy stabilizing above 80% and validation accuracy following a similar trend. Overall, the model displayed a strong learning capability, but the variations in validation accuracy suggested sensitivity to the validation dataset.

**Figure 20:**
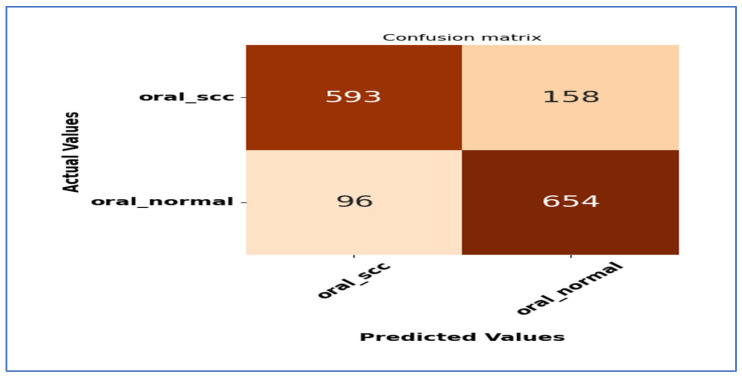

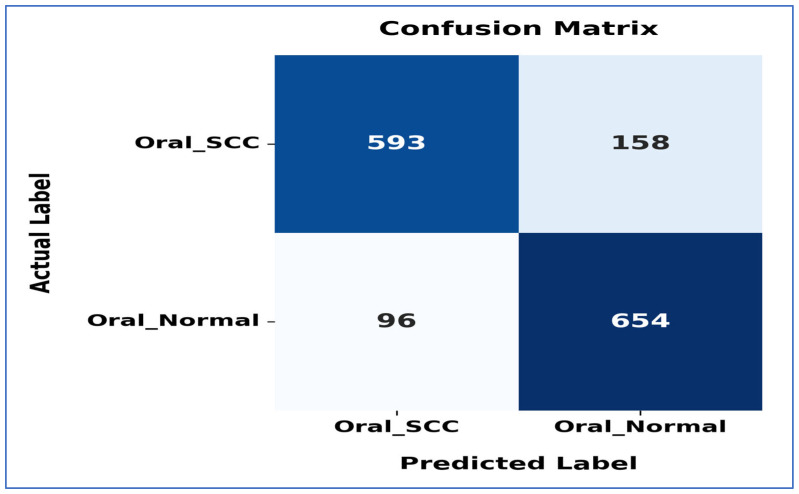
The confusion matrix for the EfficientNet-B3 model tested on the oral tumor dataset using a fixed LR.

Figure 20 shows the confusion matrix for the EfficientNet-B3 model. This matrix illustrates how well the EfficientNet-B3 model performed in distinguishing between SCC and normal cases. The model accurately classified 593 instances of the SCC class and 654 instances of the normal class. However, it misidentified 158 SCC samples as normal and 96 normal samples as SCC.

The model exhibited a higher number of FNs for the SCC class, indicating that some cancerous cases were mistakenly identified as normal. This misclassification could have serious consequences in a medical context, as failing to identify positive cases may delay diagnosis and treatment. Despite these errors, the model demonstrated reasonable performance in predicting both classes accurately, though further optimization could enhance its sensitivity to SCC cases.

### 4.4. Confidence Intervals

Confidence intervals (CIs) are essential for assessing and understanding the performance of DL models. A CI gives a range of values that likely contains the true performance measurement, like accuracy with a specified CI, usually 95%. This concept helps to quantify the uncertainty linked to predictions made by a model. It indicates whether high performance is reliable or simply due to random chance. CIs are useful for estimating how effectively a DL model can generalize to new, unseen data. A narrow CI suggests that the model performs consistently and reliably. Conversely, a wide CI may indicate potential instability in the model or a lack of sufficient data for training. Reporting CIs is essential for making model evaluations reproducible and reliable across various samples. This approach offers deeper insights compared to using point estimates by themselves. When assessing a DL classifier’s accuracy with a 95% CI, a result of 85% ± 2% suggests that the model’s actual accuracy is probably between 83% and 87%. This range provides more insight into the model’s reliability compared to simply stating the accuracy as 85% [47].

CIs play a crucial role in improving the understanding of DL models. They provide a clearer picture of model predictions, allowing better understanding of the results. By showing the range within which we expect the true values to lie, CIs bolster trust in the reliability of the findings. They offer a transparent view of the uncertainty associated with predictions, which is vital for responsible data analysis and decision making. In conclusion, CIs are an essential element of statistical analysis in DL research and its various applications [47].

**Figure 21:**
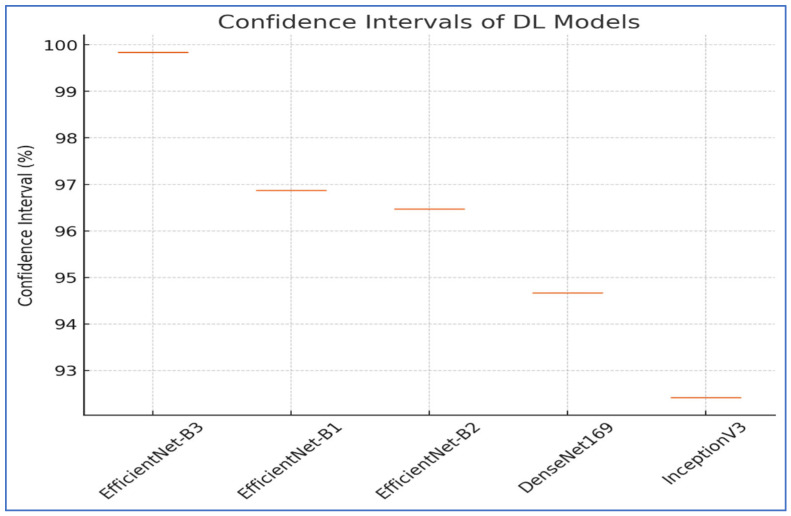
CIs for the five DL models on the test set of the oral tumor dataset.

To assess the reliability of the results from EfficientNet-B1, EfficientNet-B2, EfficientNet-B3, DenseNet169, and InceptionV3, a thorough statistical analysis was performed. This analysis concentrated on the CIs. The CIs for the five DL models are displayed in Table 11 and Figure 21 for the first experiment. The EfficientNet-B3 model displayed the highest CI range of 99.84% to 99.84%, indicating exceptional consistency in its performance. The EfficientNet-B1 model achieved a CI of 96.87% to 96.87%, demonstrating relatively lower performance compared to EfficientNet-B3. Similarly, EfficientNet-B2 had a CI of 96.47% to 96.47%, slightly lower than EfficientNet-B1. The DenseNet169 model showed a CI of 94.67% to 94.67%, which is lower than EfficientNet-B2.

Lastly, the InceptionV3 model exhibited the lowest performance, with a CI of 92.41% to 92.41%. The results emphasized that EfficientNet-B3 provided superior performance and reliability, while the other models displayed varying levels of accuracy and stability.

### 4.5. Ablation Study

An ablation study in DL is a detailed examination that assesses the significance and effect of various components or techniques within a model’s structure or training process. This approach involves deliberately removing or altering specific parts of the model to gauge their impact on overall performance. For example, in a complex CNN, an ablation study may include disabling certain layers, eliminating data preprocessing steps, or adjusting hyperparameters to see how these modifications influence metrics, like accuracy, precision, or recall.

The main objective of an ablation study is to uncover which features or design choices are vital for achieving optimal results. It assists researchers and practitioners in pinpointing unnecessary or less impactful components, improving computational efficiency, and enhancing the interpretability of models. In DL applications, such as image classification, natural language processing, or object detection, typical ablation studies might evaluate the effects of different activation functions, feature extraction layers, or attention mechanisms. For instance, removing a dropout layer from a model can demonstrate its role in mitigating overfitting.

Ablation studies are crucial for validating design choices and informing future enhancements. By offering empirical evidence regarding a model’s internal dependencies, they support the development of more robust, efficient, and understandable AI systems. The findings from these studies are often showcased in research papers through comparative tables or graphs that illustrate performance variations when certain elements are removed, making them an essential resource for furthering deep learning research and refining complex models [48].

In our research, we performed an ablation study by altering the optimizers utilized in our experiments. We began with the Adam optimizer, setting the LR to 0.0001. The outcomes demonstrated an impressive accuracy of 99.84%. Subsequently, we repeated the experiments using the Adam optimizer along with two additional optimizers, namely stochastic gradient descent (SGD) and the adaptive gradient algorithm (Adagrad). We assessed the accuracy for each class within the oral tumor dataset of the multi-cancer dataset. Additionally, we calculated the average accuracy across the two classes from the oral tumor dataset. The results, showcasing the accuracy of EfficientNet-B3 with the LRT for the three optimizers, are summarized and presented in Table 12.

Table 12 presents an analysis of the EfficientNet-B3 model’s performance on the test set of the Oral Tumor dataset using the Adagrad optimizer. For the OSCC category, the model achieved an accuracy of 98.09%, a specificity of 99.09%, and an FNR of 26.93%. The NPV was 98.92%, while precision, recall, and F1-score were 76.32%, 73.07%, and 74.66%, respectively.

In the case of normal samples, the model’s accuracy was 98.03%, with a specificity of 98.93% and an FNR of 24.50%. The NPV reached 99.02%, while precision, recall, and F1-score were 73.92%, 75.50%, and 74.70%, respectively.

Overall, across both classes, the model recorded an average accuracy of 98.06%, a specificity of 99.01%, an FNR of 25.72%, and an NPV of 98.97%. The average precision, recall, and F1-score were 75.12%, 74.28%, and 74.68%, respectively. This thorough evaluation indicates that while the model shows high accuracy and specificity, the relatively higher FNR and moderate precision and recall values highlight areas for improvement, particularly in reducing misclassification of positive cases and enhancing detection sensitivity.

Table 12 displays the performance metrics of the EfficientNet-B3 model in detecting OSCC as well as normal tissues using the SGD optimizer. For OSCC detection, the model achieved an accuracy of 98.27%, specificity of 99.12%, and an FNR of 23.07%. The NPV was 99.08%, while the precision, recall, and F1-score were 77.76%, 76.93%, and 77.35%, respectively.

In the classification of normal tissues, the model exhibited comparable performance, achieving an accuracy of 98.22%, specificity of 99.09%, and an FNR of 23.57%. The NPV was 99.06%, with precision, recall, and F1-score values of 77.05%, 76.43%, and 76.74%, respectively.

When considering the average performance across both OSCC and normal tissues, the model demonstrated an overall accuracy of 98.24%, specificity of 99.10%, and an FNR of 23.32%. The average NPV was 99.07%, while the precision, recall, and F1-score averaged 77.40%, 76.68%, and 77.04%, respectively. This analysis reveals that although the model achieved high accuracy and specificity, the metrics for recall and precision indicate that there is still potential for improvement, especially in reducing the FN rate and enhancing the balance between precision and recall.

**Figure 22:**
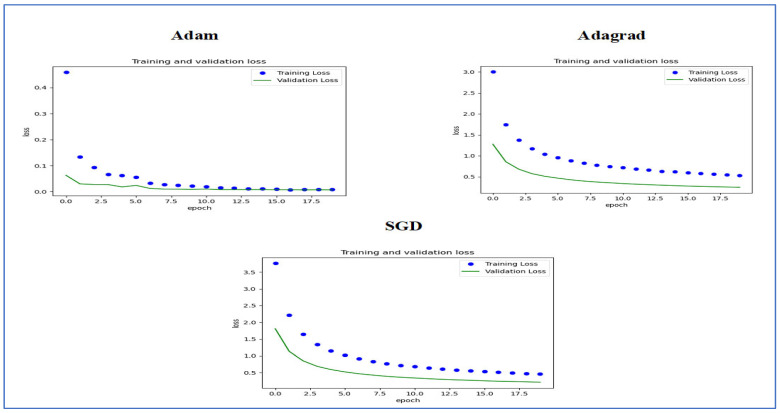
The training and validation loss for the EfficientNet-B3 model was evaluated using the LRT method with three different optimizers.

Figure 22 presents the training and validation loss curves for three optimization algorithms, namely Adam, Adagrad, and SGD. Each graph shows how the loss values evolved over 20 epochs for both the training and validation sets.

In the Adam optimizer graph, the training loss decreased consistently, beginning with a high initial value and converging quickly within the first few epochs. The validation loss exhibited a similar pattern, stabilizing with minimal deviation from the training loss, which indicates effective generalization performance.

The Adagrad optimizer displayed a slower initial decrease in training loss compared to Adam. Nevertheless, it also achieved convergence throughout the training epochs, albeit with a slightly higher final loss. The validation loss showed a comparable downward trend but remained slightly above that of Adam.

For the SGD optimizer, both training and validation loss started at higher values than the other optimizers. The decrease in loss was more gradual; despite notable improvements, the final loss was still higher than those recorded with Adam and Adagrad. The difference between training and validation loss was more significant, suggesting possible issues with overfitting or less efficient learning.

In summary, the Adam optimizer exhibited the best performance in reducing loss and achieving stable training and validation curves, followed by Adagrad. SGD demonstrated slower convergence and relatively higher losses, highlighting the performance differences among these optimizers for the training and validation sets used.

**Figure 23:**
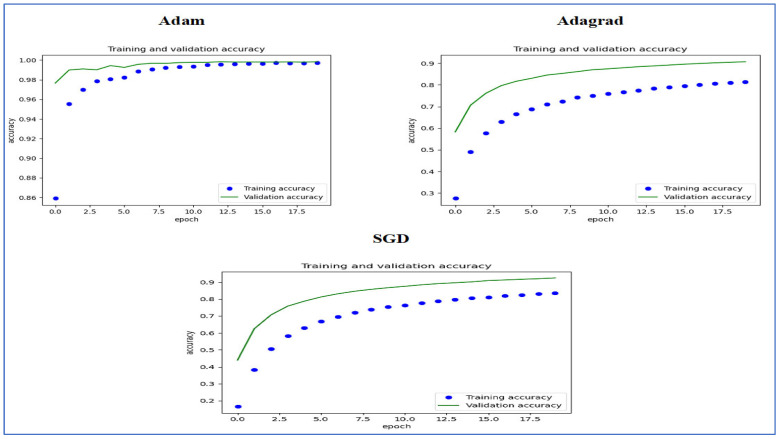
The training and validation accuracy for the EfficientNet-B3 model was evaluated using the LRT method with three different optimizers.

Figure 23 shows the training and validation accuracy over 18 epochs for the EfficientNet-B3 model, which was utilized to classify two categories using the LRT method and three different optimizers, namely Adam, Adagrad, and SGD.

For the Adam optimizer, the training accuracy increased rapidly at first and then stabilized around 1.0, indicating effective learning. The validation accuracy also rose initially but leveled off slightly lower than the training accuracy. The model exhibited strong performance on both training and validation data.

For the Adagrad optimizer, the training accuracy quickly stabilized around 0.8, with the validation accuracy following a similar trend and plateauing at a slightly lower level. The small gap between training and validation accuracy suggested minimal signs of overfitting, indicating that the model generalizes well to new data.

For the SGD optimizer, the training accuracy stabilized around 0.85, while the validation accuracy plateaued slightly lower. The slight gap between the two accuracy curves hinted at a potential for minor overfitting, although the model still demonstrated strong generalization to new data. Overall, it showed solid performance on both training and validation datasets. In conclusion, all optimizers resulted in strong model performance with minimal overfitting, making the model a suitable candidate for deployment.

### 4.6. Comparing the Results with the State-of-the-Art

OSCC is the sixth most common cancer worldwide, and its incidence is increasing. The diagnosis of OSCC mainly relies on HIs, but this approach can be time-consuming, costly, and requires specialized skills. Manual diagnosis often results in inaccuracies and inconsistencies, underscoring the critical need for automated and reliable diagnostic methods to improve early detection and treatment outcomes.

The previously mentioned research used a fixed LR, while our proposed model implemented the LRT to overcome the limitations of a fixed LR. The LRT dynamically adjusts the LR during training based on various factors, such as gradient magnitudes, loss convergence, and training progress. This flexibility allows the model to navigate complex optimization landscapes more efficiently. By adapting the LR in real time, the LRT facilitates faster convergence, accelerating progress during the initial training phases and reducing oscillations or overshooting as optimization approaches convergence. Consequently, our proposed model achieved higher accuracy compared to the current state-of-the-art approaches in this field.

We carried out an experiment that examined the use of transfer learning by pre-training the models EfficientNet-B1, EfficientNet-B2, EfficientNet-B3, DenseNet169, and InceptionV3. This was achieved using the multi-cancer dataset, which includes the oral tumor dataset. The dataset is organized as follows: 70% of the data is used for the training set (6998 histopathological images or HIs), while 15% is allocated for the test set (1501 HIs), and the remaining 15% is set aside for the validation set (1501 HIs).

During the first stage of transfer learning, we performed supervised pre-training on these five DL models using the ImageNet dataset. In the subsequent stage, we improved the models further by using the training set of the oral tumor dataset. When tested on the oral tumor dataset, the model showed excellent results, achieving an accuracy of 99.84% and a specificity of 99.92%, along with other strong performance metrics. These results suggest that the model could simplify the diagnostic process, reduce costs, and improve patient outcomes in clinical environments.

**Figure 24:**
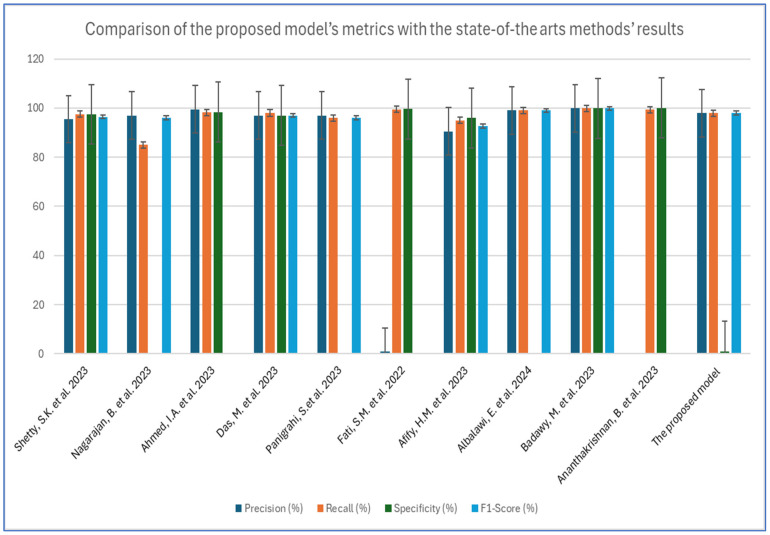
Comparison of the proposed model’s metrics with the state-of-the arts methods’ results [3,8,14,17,34,35,36,37,38,39].

Table 13 and Table 14, along with Figure 24, provide a comparative analysis of different methods for detecting OSCC tumors, focusing on their accuracy and the datasets used. Shetty, S.K. et al. [3] implemented the DPODTL-OSC3 model, achieving an accuracy of 97.28% on the oral tumor dataset. Nagarajan, B. et al. [17] used a modified gorilla troops optimizer, reaching an accuracy of 95%. Ahmed, I.A. et al. [34] employed fused CNNs, which yielded the highest accuracy of 99.3%. Das, M. et al. [14] adopted a CNN-based model, reporting an accuracy of 97.82%. Panigrahi, S. et al. [35] utilized a DCNN model, achieving an accuracy of 96.6%. Fati, S.M. et al. [36] who combined hybrid features from AlexNet, DWT, LBP, FCH, and GLCM in an ANN model, achieved an accuracy of 99.1%. Afify, H.M. et al. [37] integrated ResNet-101 and EfficientNet-B0 with Grad-CAM, with ResNet-101 achieving 100% accuracy at 100× magnification, and EfficientNet-B0 reaching 95.65% at 400× magnification. Albalawi, E. et al. [38] applied EfficientNet-B3, resulting in an accuracy of 99%.

Furthermore, Badawy, M. et al. [8] combined AO, GTO, and DenseNet201, recording accuracies of 99.25% with AO and 97.27% with GTO. Ananthakrishnan, B. et al. [39] combined CNN with a RF classifier, reporting an impressive accuracy of 99.65%.

Lastly, the proposed model using EfficientNet-B3 and LRT demonstrated exceptional performance with an accuracy of 99.84% at magnifications of both 100× and 400×, making it one of the top-performing methods for oral tumor classification on the same dataset.

## 5. Conclusions

This paper presented an optimized model based on EfficientNet-B3, designed to classify HIs into two categories, namely normal tissue and OSCC. The model utilized feature extraction with pre-trained weights from five different architectures, namely EfficientNet-B1, EfficientNet-B2, EfficientNet-B3, DenseNet169, and InceptionV3. A novel LRT dynamically modifies the learning rate at the beginning of each epoch by analyzing accuracy and loss trends from prior epochs, thereby increasing training efficiency. The goal of the proposed model is to enhance the early detection of OSCC, potentially decreasing both diagnostic time and costs for healthcare providers.

We conducted two experiments. In the first experiment, we incorporated the LRT into our training process, while the second experiment utilized a constant LR algorithm. Both experiments employed transfer learning. The experiments were performed using the oral tumor subset of the multi-cancer dataset, which consists of 1224 HIs of normal and OSCC tissues captured at magnifications of 100× and 400×. Preprocessing steps included data augmentation (to enhance sample diversity), resizing (for model compatibility), and normalization. The fine-tuned classifier demonstrated superior performance compared to conventional models in comparative analyses.

We evaluated the performance of our proposed DL model against traditional models. The results showed impressive metrics, with accuracy at 99.84%, specificity at 99.92%, recall at 97.93%, precision at 97.94%, and an F1-score of 97.93%. The evaluation of our classifier indicated that it offers exceptional tuning results compared to other recent DL models. However, the running time of our proposed system remains a limitation. Future work will aim to enhance the forecasting capabilities of the proposed structure through further improvements and feature identification.

## Figures and Tables

**Table 1:** The two classes of HIs in the oral tumor dataset.

Resolution	Type	Image Count
100×	Normal	89
100×	OSCC	439
400×	Normal	201
400×	OSCC	495
Total		1224

**Table 2:** The hyperparameter values used in the two experiments.

Parameter	Value
img_size	224 × 224
No. of epochs	30
Batch size	115
Activation	softmax
B normalization	momentum = 0.99 and epsilon = 0.001
kernel_regularizer	l2 (l = 0.016)
activity_regularizer	l1(0.006)
Optimizer	Adama
Initial learning rate	0.0001
Patience	10

**Table 3:** The results of five DL models on the test set of the oral tumor dataset using the LRT.

Dataset	Model	Accuracy (%)	Specificity (%)	FNR (%)	NPV (%)	Precision (%)	Recall (%)	F1-Score (%)
Oral tumor	EfficientNet-B3	99.84	99.92	2.07	99.92	97.94	97.93	97.93
	EfficientNet-B1	96.87	96.87	3.13	96.87	96.87	96.87	96.87
	EfficientNet-B2	96.47	96.47	3.53	96.47	96.47	96.47	96.47
	DenseNet169	94.67	94.67	5.33	94.71	94.71	94.67	94.67
	InceptionV3	92.41	92.41	7.59	92.41	92.41	92.41	92.41

**Table 4:** The results of EfficientNet-B3 model on the test set of the oral tumor dataset using the LRT.

Model	Class	Accuracy (%)	Specificity (%)	FNR (%)	NPV (%)	Precision (%)	Recall (%)	F1-Score (%)
EfficientNet-B3	OSCC	99.84	99.90	1.73	99.93	97.62	98.27	97.94
	Normal	99.84	99.93	2.40	99.90	98.26	97.60	97.93
	Average	99.84	99.92	2.07	99.92	97.94	97.93	97.93

**Table 5:** The results of EfficientNet-B1model on the test set of the oral tumor dataset using the LRT.

Model	Class	Accuracy (%)	Specificity (%)	FNR (%)	NPV (%)	Precision (%)	Recall (%)	F1-Score (%)
EfficientNet-B1	OSCC	96.87	96.67	2.93	97.05	96.68	97.07	96.88
	Normal	96.87	97.07	3.33	96.68	97.05	96.67	96.86
	Average	96.87	96.87	3.13	96.87	96.87	96.87	96.87

**Table 6:** The results of EfficientNet-B2 DL model on the test set of the oral tumor dataset using the LRT.

Model	Class	Accuracy (%)	Specificity (%)	FNR (%)	NPV (%)	Precision (%)	Recall (%)	F1-Score (%)
EfficientNet-B2	OSCC	96.47	96.27	3.33	96.65	96.29	96.67	96.48
	Normal	96.47	96.67	3.73	96.29	96.65	96.27	96.46
	Average	96.47	96.47	3.53	96.47	96.47	96.47	96.47

**Table 7:** The results of DenseNet169 DL model on the test set of the oral tumor dataset using the LRT.

Model	Class	Accuracy (%)	Specificity (%)	FNR (%)	NPV (%)	Precision (%)	Recall (%)	F1-Score (%)
DenseNet169	OSCC	94.67	96.13	6.79	93.39	96.02	93.21	94.59
	Normal	94.67	93.21	3.87	96.02	93.39	96.13	94.74
	Average	94.67	94.67	5.33	94.71	94.71	94.67	94.67

**Table 8:** The results of InceptionV3 model on the test set of the oral tumor dataset using the LRT.

Model	Class	Accuracy (%)	Specificity (%)	FNR (%)	NPV (%)	Precision (%)	Recall (%)	F1-Score (%)
InceptionV3	OSCC	92.41	92.67	7.86	92.18	92.64	92.14	92.39
	Normal	92.41	92.14	7.33	92.64	92.18	92.67	92.42
	Average	92.41	92.41	7.59	92.41	92.41	92.41	92.41

**Table 9:** The results of EfficientNet-B3 model on the test set of the oral tumor dataset using a fixed LR.

Model	Class	Accuracy (%)	Specificity (%)	FNR (%)	NPV (%)	Precision (%)	Recall (%)	F1-Score (%)
EfficientNet-B3	OSCC	83.08	87.20	21.04	80.54	86.07	78.96	82.36
	Normal	83.08	78.96	12.80	86.07	80.54	87.20	83.74
	Average	83.08	83.08	16.92	83.30	83.30	83.08	83.05

**Table 10:** A comparative analysis of EfficientNet-B3 on the test set of the oral tumor dataset using the LRT and a fixed LR.

Model	Class	Accuracy (%)	Specificity (%)	FNR (%)	NPV (%)	Precision (%)	Recall (%)	F1-Score (%)
LRT	OSCC	99.84	99.90	1.73	99.93	97.62	98.27	97.94
	Normal	99.84	99.93	2.40	99.90	98.26	97.60	97.93
	Average	99.84	99.92	2.07	99.92	97.94	97.93	97.93
Fixed LR	OSCC	83.08	87.20	21.04	80.54	86.07	78.96	82.36
	Normal	83.08	78.96	12.80	86.07	80.54	87.20	83.74
	Average	83.08	83.08	16.92	83.30	83.30	83.08	83.05

**Table 11:** The CIs of the five DL models on the test set of the oral tumor dataset.

DL Model	CI (%)
EfficientNet-B3	[99.84, 99.84]
EfficientNet-B1	[96.87, 96.87]
EfficinetNet-B2	[96.47, 96.47]
DenseNet169	[94.67, 94.67]
InceptionV3	[92.41, 92.41]

**Table 12:** Average results of EfficientNet-B3 with the Adam, Adagrad, and SGD optimizers on the test set using the LRT.

Dataset	Model	Accuracy (%)	Specificity (%)	FNR (%)	NPV (%)	Precision (%)	Recall (%)	F1-Score (%)
Adam	OSCC	99.84	99.90	1.73	99.93	97.62	98.27	97.94
	Normal	99.84	99.93	2.40	99.90	98.26	97.60	97.93
	Average	99.84	99.92	2.07	99.92	97.94	97.93	97.93
Adagrad	OSCC	98.09	99.09	26.93	98.92	76.32	73.07	74.66
	Normal	98.03	98.93	24.50	99.02	73.92	75.50	74.70
	Average	98.06	99.01	25.72	98.97	75.12	74.28	74.68
SGD	OSCC	98.09	99.09	26.93	98.92	76.32	73.07	74.66
	Normal	98.03	98.93	24.50	99.02	73.92	75.50	74.70
	Average	98.06	99.01	25.72	98.97	75.12	74.28	74.68

**Table 13:** Comparison of the proposed model’s results with recent models’ results.

Reference	Methodology	Accuracy	Dataset
Shetty, S.K. et al. [3]	DPODTL-OSC3	97.28%	Oral tumor
Nagarajan, B. et al. [17]	Modified gorilla troops optimizer	95%	Oral tumor
Ahmed, I.A. et al. [34]	Fused CNNs	99.3%	Oral tumor
Das, M. et al. [14]	CNN model	97.82%	Oral tumor
Panigrahi, S.et al. [35]	DCNN model	96.6%,	Oral tumor
Fati, S.M. et al. [36]	ANN model utilizing hybrid features from AlexNet, along with DWT, LBP, FCH, and GLCM	99.1%	Oral tumor
Afify, H.M. et al. [37]	ResNet-101, EfficientNet-B0, and Grad-CAM	ResNet-101:100% at 100× and 92.75% at 400×. EfficientNet-B0: 94.34% at 100× and 95.65% at 400× magnification	Oral tumor
Albalawi, E. et al. [38]	EfficientNet-B3	99%	Oral tumor
Badawy, M. et al. [8]	AO, GTO, and DenseNet201	99.25% with the AO and 97.27% with the GTO.	Oral tumor
Ananthakrishnan, B. et al. [39]	CNN+RF	99.65%	Oral tumor
The proposed model	EfficientNet-B3 and LRT	99.84% at 100× magnification and 400× magnification	Oral tumor

**Table 14:** Comparison of the proposed model’s metrics with the state-of-the arts methods’ results.

Reference	Precision (%)	Recall (%)	Specificity (%)	F1-Score (%)
Shetty, S.K. et al. [3]	95.51	97.46	97.46	96.43
Nagarajan, B. et al. [17]	97	85		96
Ahmed, I.A. et al. [34]	99.5%	98.2%	98.35	
Das, M. et al.[14]	97	98	97	97
Panigrahi, S.et al. [35]	97	96		96
Fati, S.M. et al. [36]	99.71	99.5	99.61	
Afify, H.M. et al. [37]	90.48	95	95.92	92.68
Albalawi, E. et al. [38]	99	99		99
Badawy, M. et al. [8]	99.86	99.86	99.86	99.86
Ananthakrishnan, B. et al. [39]		99.3	100	
The proposed model	97.94	97.93	99.92%	97.93

## Data Availability

We utilized the oral tumor dataset, which is a subset of the multi-cancer dataset. The multi-cancer dataset can be found on Kaggle at the following link: Multi-Cancer Dataset (Accessed in 2024). The oral tumor dataset is also available on Kaggle at the following link: Oral Tumor Dataset (Accessed in 2021).
